# Dynamics of CD44^+^ bovine nucleus pulposus cells with inflammation

**DOI:** 10.1038/s41598-024-59504-7

**Published:** 2024-04-21

**Authors:** J. R. Ferreira, J. Caldeira, M. Sousa, M. A. Barbosa, M. Lamghari, G. Almeida-Porada, R. M. Gonçalves

**Affiliations:** 1 I3S—Instituto de Investigação e Inovação em Saúde, Universidade Do Porto, Porto, Portugal; 2 INEB—Instituto de Engenharia Biomédica, Universidade Do Porto, Porto, Portugal; 3 Instituto de Ciências Biomédicas Abel Salazar, Universidade Do Porto, Porto, Portugal; 4 WFIRM—Wake Forest Institute for Regenerative Medicine, Winston-Salem, North Carolina USA; 5 Present Address: Cell & Gene Therapy Safety, Clinical Pharmacology & Safety Science, R&D, AstraZeneca, Molndal, Sweden

**Keywords:** Inflammation, Intervertebral disc, CD44, Proteomics, IL-1β, Biological techniques, Regenerative medicine, Cartilage

## Abstract

Intervertebral Disc (IVD) degeneration has been associated with a chronic inflammatory response, but knowledge on the contribution of distinct IVD cells, namely CD44, to the progression of IVD degeneration remains elusive. Here, bovine nucleus pulposus (NP) CD44 cells were sorted and compared by gene expression and proteomics with the negative counterpart. NP cells were then stimulated with IL-1b (10 ng/ml) and dynamics of CD44 gene and protein expression was analyzed upon pro-inflammatory treatment. The results emphasize that CD44 has a multidimensional functional role in IVD metabolism, ECM synthesis and production of neuropermissive factors. CD44 widespread expression in NP was partially associated with CD14 and CD45, resulting in the identification of distinct cell subsets. In conclusion, this study points out CD44 and CD44-based cell subsets as relevant targets in the modulation of the IVD pro-inflammatory/degenerative cascade.

## Introduction

Understanding the inflammatory cascade involved in intervertebral disc (IVD) degeneration is crucial for the development of more sophisticated immunomodulatory therapies that could contribute to the recovery of the disc’s native function. IVD degeneration is the most common cause of low back pain (LBP), and a major hurdle for rehabilitation services worldwide^1^. Discogenic pain is the pain associated with IVD degeneration, without apparent nerve compression, that has been strongly associated with inflammation. Despite anti-inflammatory treatments that partially reduce pain, the restoration of a healthy IVD has not yet been attained.

During IVD degeneration, an imbalance of the activity of nucleus pulposus (NP) cells occurs^2^, leading to extracellular matrix (ECM) remodelling and loss of tissue hydration, which culminates in failure of disc integrity/function. Underlying this process is the intense production of pro-inflammatory mediators whose ability to modulate most steps of the IVD degenerative cascade have been well documented^2,3^. In general, IVD cells are known to produce pro-inflammatory factors which then promote the recruitment of immune cells, namely macrophages^2^. But, while evidence for cellular diversity in the IVD keeps increasing, the specific contribution of the different IVD cell types to the physiology and pathology of IVD degeneration remains unknown. IVD cells have been divided into subpopulations with distinct responsiveness to differentiation stimuli^4^, and a unique contribution to the production of healthy ECM^5^. For example, cells capable of clonal expansion and multi-lineage differentiation have been isolated from the IVD, with different yields and sets of defining markers (both notochordal and mesenchymal stem cell marker sets)^6^. More recently, the possibility of existing resident macrophage-like cells in the IVD has been suggested^7^. Murine F4/80^+^ (macrophage marker) cells derived from injured IVD tissue were shown to respond to a traumatic stimulus differently than recruited bone-marrow derived F4/80^+^ cells^8^, distinctly contributing to the inflammatory IVD microenvironment. Nevertheless, the IVD cell players that are involved in the inflammatory cascade remain to be determined.

One of the few IVD markers more widely reported as an inflammatory mediator associated with IVD degeneration is CD44. CD44 has been described as a marker of notochordal cells in rat IVD development^9^, associated with IVD progenitor cells in human embryogenesis^10^ and also pointed out as a specific marker of NP cells^11^. This cell surface marker is the main ligand for hyaluronic acid (HA)^12^, one of the most abundant glycosaminoglycans (GAGs) in the IVD’s ECM^13^, and a factor known to regulate production of other ECM components, such as collagen^14^, laminin, fibronectin, and aggrecan^15^. In parallel, CD44 has been described as a key pro-inflammatory regulator in response to several factors including IL-1β and TNF-α, the most described key players of the IVD inflammatory cascade^16^.

In this work, we propose to investigate the response of CD44-expressing cells to IL-1β–mediated IVD degeneration, using a standardized pro-inflammatory/degenerative NP organotypic model of bovine origin. This model was previously shown to closely mimic the initial degenerative IVD catabolic cascade, with the increase of pro-inflammatory cytokines (*IL-6*, *IL-8*, *TNF-α* and PGE_2_), accompanied by an upregulation of matrix degrading enzymes (*MMP1* and *MMP3*) and a significant downregulation of matrix components (*COL2A1* and *ACAN*)^17^. Importantly, the use of bovine tissue assures that the cellular content being analysed is relevant to the human context, given the very low frequency of distinct progenitor notochordal cells in adults from both species, the biomechanics, the disc morphology and the ECM biochemical composition^18^.

We performed proteomic analysis of bovine CD44^+^ NP cells, unveiling a novel functional role of this cell subset in the IVD. Moreover, we investigated the dynamics of CD44 expression in response to IL-1β, particularly its association with co-expression of CD14 (a monocyte/macrophage lineage marker) and CD45 (a pan-hematopoietic marker), offering novel insights into the complex cellular dynamics mediating the catabolic cascade in IVD, which could open new horizons to develop novel targeted therapies in this joint.

## Materials and methods

### Fluorescent activated cell sorting (FACS) of NP cells

Bovine tails were purchased at the Carnes Landeiro SA slaughterhouse, with the ethical approval of Portuguese Agency for Animal Welfare (Direção-Geral de Alimentação e Veterinária, DGAV). IVDs were isolated from the tails of young animals (1 year old) up to 3 h post-slaughter and cultured, as previously described^17^. Briefly, discs were dissected from the surrounding tissue with a scalpel and the NP center portion was collected with a 9 mm-puncher. For FACS of the disc cells based on the expression of CD44, fresh NP was minced into small pieces with a scalpel before enzymatic digestion with 0.2% collagenase type IA (Sigma) in DMEM Low Glucose supplemented with 1% P/S and 0,5% Amphotericin B, at 37 °C, overnight, with magnetic agitation. NP cells were then passed through a 100 μm cell strainer and resuspended in PBS + 2% FBS (Capricorn) + 0.1% sodium azide (sigma) (FACS buffer), before incubation with an APC-conjugated CD44 antibody (1:400, BioLegend, clone IM7) for 15 min, at room temperature. After the first 10 min, the Fixable Viability Stain 780 (1:1000, BD Horizon) was added to the incubation mix for the remaining 5 min to guarantee posterior exclusion of dead cells. Cells were then washed two times in PBS 1 × and run on FACSAria II sorter (Becton Dickinson), under mild sorting conditions: 100-μm nozzle, sheath pressure of 30 pounds per square inch (PSI) and an acquisition rate of 2000–3000 events per second. The purity of sorted fractions was confirmed by FACS reanalysis. Cell collection tubes were prepared according to the analysis being performed after. For proteomic analysis, cells were collected to 1.5 mL Protein LoBind tubes (Eppendorf) with 200μL of sterile PBS 1x. For such experiments, the FACS buffer normally used was substituted by PBS 1x, without FBS, to avoid the contamination of the samples with the bovine proteins from the serum. For gene expression analysis and cytospinning, cells were collected to 1.5 mL centrifuge tubes with 200μL of sterile PBS 1x. The sorting experiment was analyzed using the FACSDiva Software (Becton Dickinson).

### Imaging flow cytometry analysis of NP cells

NP CD44^+^ and CD44^-^ cells were analysed by imaging flow cytometry. NP cells obtained upon digestion were labelled with an FITC-conjugated CD44 antibody (1:50, AbD Serotec, clone IL-A118) as described for cell sorting and with the nuclear DRAQ5™ fluorescent probe (1:500, eBioscience) for accurate detection of the cells in stream. Cells were run on ImageStream^X^ (Millipore) and analysed with IDEAS software for cell morphological features such as circularity, aspect ratio, thickness, length and area, using the masks provided by the software.

### NP cells gene expression analysis

Total RNA was extracted, and reverse transcribed into cDNA for gene expression analysis both from discs cultured in the organotypic model or cells sorted from fresh discs. For cultured discs, the NP tissue was enzymatically digested for 1 h with 2 mg/mL protease from Streptomyces griseus (Sigma-Aldrich) in DMEM, under agitation (50 rpm), at 37 °C. Digestion was inhibited by the addition of FBS (10 μL/mL) (Capricorn Scientific) and the remaining tissue was washed twice with cold PBS 1x. The tissue pellet was then resuspended in TRIzol Reagent (Invitrogen), frozen with liquid nitrogen and stored at −80 °C until posterior analysis. Sorted cells were centrifuged the pellet was also stored at −80 °C in TRIzol Reagent (Invitrogen). The RNA extraction protocol recommended by the manufacturer was followed for the sorted cells. However, for the cultured discs, it was only respected up to the collection of the aqueous phase containing the RNA. In this case, the aqueous phase was removed, mixed with isopropanol, and transferred to the binding columns of the ReliaPrep RNA Cell Miniprep System (Promega), where it was purified according to manufacturer’s instructions. RNA was quantified using a NanoDrop spectrophotometer (Infinite M200; Tecan) and RNA quality was confirmed by determining the RNA integrity ratio in a Bioanalyzer, using the RNA 6000 Pico Kit.

Reverse transcription of the NP cells’ total RNA was performed using the SuperScript III RT Kit (Invitrogen) completed with deoxynucleotide mix (10 mM) and random hexamer primers (50 μM). The obtained cDNA was used for qRT-PCR. Specific primer pairs for bovine *CD44, CD14, IL-6, IL-8, MMP3, VEGFA, TIMP1, TIMP2, ADAMTS5, COL2A1*, *ACAN, FoxF1, Gal3* and *GAPDH*, were purchased from Invitrogen (sequences available in Table S1). qRT-PCR mixes were prepared with iTaq Universal SYBR Green Supermix (Bio-Rad), and ran in a CFX96 Real-Time qPCR System (Bio-Rad). Gene expression was considered valid for Cq < 35. The 2-∆Cq/2-∆∆Cq analysis method was used to compare relative expression levels of the transcribed genes. Briefly, the average Cq of each sample was normalized to the respective Cq of the reference gene GAPDH (∆Cq) for direct comparison between sorted populations or further normalized to the control sample relative to the organotypic model experiments (∆∆Cq). Final results are shown as 2-∆Cq/2-∆∆Cq. Expression of *KRT8, KRT18, Pax1, TBXT, CD24, Tie2* and *CD45* was also assessed by qRT-PCR, but very low expression (Ct > 35) was detected.

### NP cells proteomic analysis

NP cells isolated from fresh bovine tails (male cows, 1 year old, cross-breed) and posteriorly sorted based on the expression of CD44, were collected in sterile PBS. After centrifugation, the cell pellet was resuspended in Radioimmunoprecipitation assay (RIPA) lysis buffer supplemented with a cocktail of protease inhibitors (10 μl/mL Phenylmethylsulfonyl Fluoride, 10 μl/mL Sodium Fluoride, 10 μl/mL Leupeptin, 10 μl/mL Aprotinin, 20 μl/mL Sodium Orthovanadate and 50 μl/mL Sodium Pyrophosphate) and incubated for 30 min at 4 °C, to promote cell lysis. Lysates were then centrifuged at 14,000 rpm for 10 min at 4 °C to clear the protein-containing supernatant from the remaining cell debris. Total protein content was then quantified with the DC Protein Assay kit (Bio-Rad), according to manufacturer’s instructions, and 10–30 ug of total protein were used for proteomic analysis.

The identification and quantification of proteins was performed by nano liquid chromatography-tandem mass spectrometry (LC–MS/MS), using the Ultimate 3000 liquid chromatography system coupled with a Q-Exactive Hybrid Quadrupole-Orbitrap mass spectrometer (Thermo Scientific). Samples were separated using a nano-C18 column at 300 nL/min with the data acquisition controlled by the Xcallibur and Tune software (Thermo Scientific). The mass spectrometer was operated in data-dependent positive acquisition mode alternating between a full scan (m/z 380–1580) and subsequent HCD MS/MS of the 10 most intense peaks from full scan. The mass spectrometry proteomics data have been deposited to the ProteomeXchange Consortium via the PRIDE partner repository with the dataset identifier PXD048373.

The mass spectra detected was initially processed and visualized with the Proteome Discoverer software (v 2.5, Thermo Scientific) and the results obtained were compared against the UniProt database (Bos Taurus) to identify the proteins detected, as previously optimized by our group^18,19^. An independent False Discovery Rate (FDR) analysis provided by the software was set at 1% as a first identification quality filter, with proteins considered by the software to have been detected with less than a high confidence regarding this parameter excluded from further analysis. Proteins determined to be technical contaminants (list available at the MaxQuant software (www.maxquant.org) including cytoskeletal, hair, epidermal keratin, trypsin and LysC) were also excluded. Due to the high biological variability associated with these samples, an extra set of filters had to be established to guarantee experimental confidence. Thus, only proteins that were detected in all 4 biological replicates with high identification confidence for at least three of the replicates in at least one of the conditions (CD44^+^ vs CD44^-^ cells) were considered. The group of proteins therein derived was established as the background for posterior gene ontology analysis. Moreover, as samples were obtained, processed, and analyzed in two different experimental sets, with two replicates per experiment, and some experimental bias was easily detected, only proteins whose abundance ratio between CD44^+^ and CD44^-^ cells was consistent throughout at least 3 of the 4 samples were considered biologically altered between the two conditions. The list of proteins with consistently altered abundances was then subjected to a multiple t test statistical analysis performed using the Graphpad Prism software (v 6.01) to ascertain the statistical significancy of the tendencies observed. Lastly, a differential expression cut-off ratio > 1.5 and < 0.77 between CD44^+^ and CD44^-^ cells was considered for functional pathway analysis. The resultant significantly altered proteins were subjected to a Principal Component Analysis and analyzed using the Functional Annotation Clustering Tool from the DAVID Database (v 6.8) for their significant enrichment in specific clusters of gene ontology terms relatively to the detected list of background proteins. No clusters of gene ontology terms were found to be significantly enriched amongst the proteins significantly altered between conditions. An analysis of all the functional pathways affected by the significantly altered proteins was also performed using the Reactome software (v 76).

### Pro-inflammatory/degenerative Bovine NP Organ Culture

Bovine NP explants obtained as described for cell sorting were maintained in culture under 0.46 MPa static loading applied on cell culture inserts placed on top of the discs, in 6-well plates, as previously reported^17^. The medium composition is based on DMEM Low glucose medium supplemented with 5% FBS, 1% P/S, 0.5% Amphotericin B and with osmolarity adjusted to IVD-physiological 400 mOsm by addition of 1.5% NaCl/KCl (5 M/0.4 M). Culture conditions were kept at 37 °C under reduced oxygen atmosphere (6% O_2_, 8.5% CO_2_) and saturated humidity with medium changes at every two days. Five days after dissection, degeneration was induced with a 21G needle puncture and culture medium supplementation with 10 ng/mL of human IL-1β (PeproTech). Organotypic culture was then maintained for 2, 7 or 14 days before sample processing. Non-stimulated control experiments were maintained. For longer experiments (7 or 14 days), cell culture medium (without IL-1β supplementation) was exchanged (25%) each 4/5 days.

### Flow cytometry analysis of CD44 in NP cells

CD44 expression in NP cells obtained from organ cultures was analyzed by flow cytometry, similarly to what has been described for cell sorting. Briefly, after tissue digestion, NP cells were stained with an APC-conjugated CD44 antibody (1:400, BioLegend, clone IM7) for 15 min, at room temperature. After the first 10 min, the Fixable Viability Stain 780 (1:1000, BD Horizon) was added to the incubation mix for the remaining 5 min to guarantee posterior exclusion of dead cells. Cells were then washed two times in PBS 1 × before fixation in PFA 1%. After filtration through a cell straining mesh, a single-cell suspension was run in a FACSCanto II (Beckton Dickinson) equipment and results were analyzed with FlowJo software, version X. Unstained and isotype-stained samples were used as controls.

### Immunofluorescence staining for CD44 and co-localization analysis with CD14 and CD45

Expression of CD44, CD45 and CD14 in the NP was ascertained by immunofluorescence staining performed on tissue derived from the organotypic model. Discs were embedded in OCT cryostat sectioning medium, snap frozen in liquid nitrogen-chilled 2-methylbutane before and stored at −20 °C before further processing. OCT-embedded cryopreserved discs were cut into 50 μm thick sections using a cryostat. Sections were then fixed with cold methanol for 15 min and stored at −20 °C before proceeding for the staining protocol. For the immunofluorescence staining, slides were brought to room temperature for 30 min before sample permeabilization with PBS + 0.5% Tween-20 for 30 min at room temperature. Samples were then blocked with a Serum Free, Protein Block solution (Dako), for 1 h at room temperature. Following three washing steps with PBS + 0.5% Tween-20, tissue sections were incubated with the primary antibodies: mouse anti-CD14 (1:200, Bio-Rad, clone CC-G33), rabbit anti-CD45 (1:50, Bioss, polyclonal) and rat anti-CD44 (1:50, Thermofisher, clone Hermes-1), diluted in Antibody Diluent with Background Reduction Solution (Dako) overnight at 4 °C in a humid environment. Samples were washed three times with PBS + 0.5% Tween-20 and incubated with AF488-donkey anti-rat, AF594-goat anti-rabbit and AF647-goat anti-mouse (1:500, Jackson ImmunoResearch) secondary antibodies and 4’, 6-Diamidino-2-Phenylindole, dilactate (1:1000, DAPI, Invitrogen) for 1 h at room temperature, in the dark. Samples were then washed, mounted, sealed and stored at 4 °C until further analysis. Control conditions were prepared for each sample type by repeating the protocol with secondary antibodies. All samples were stained at the same time. For the confirmation of markers’ expression at the membrane level, the same protocol without Tween-20 was also performed. Representative images covering one third of the total area were recorded using a laser scanning confocal microscope (SP8, Leica), with the same exposure time for all samples. Quantification of the percentage of cells expressing any of the three markers was done automatically using a custom-made ImageJ (Wayne Rasband, NIH, v 1.53c). The script was created to: (1) detect cells based on nuclear staining; (2) determine a region of interest (ROI) around each nucleus; and (3) quantify the average fluorescence intensity inside that ROI for each channel. The intensity threshold above which cells were considered positive for each marker was determined according to the average intensity found in the negative control.

### Statistical analysis

Results are presented in box and whiskers plots, as median interquartile range (IQR) or bar charts. The data from this work, except for the proteomic analysis results, did not pass the normality test (D’Agostine and Pearson normality test), and, as such, nonparametric tests were applied. When two groups were compared for multiple timepoints, unpaired multiple t tests were performed, with the Holm-Sidak multiple comparison test as post hoc. When only two groups were directly compared, statistical analysis was performed using non-parametric analysis with Wilcoxon test for paired values, or unpaired Mann–Whitney test, for unpaired values. A confidence level of at least 95% (*p* < 0.05) was set. Graph Pad Prism v6.01 software was used for the analysis.

## Results

### CD44^+^ and CD44^***-***^ bovine NP cells present distinct gene expression profile

**Figure 1:**
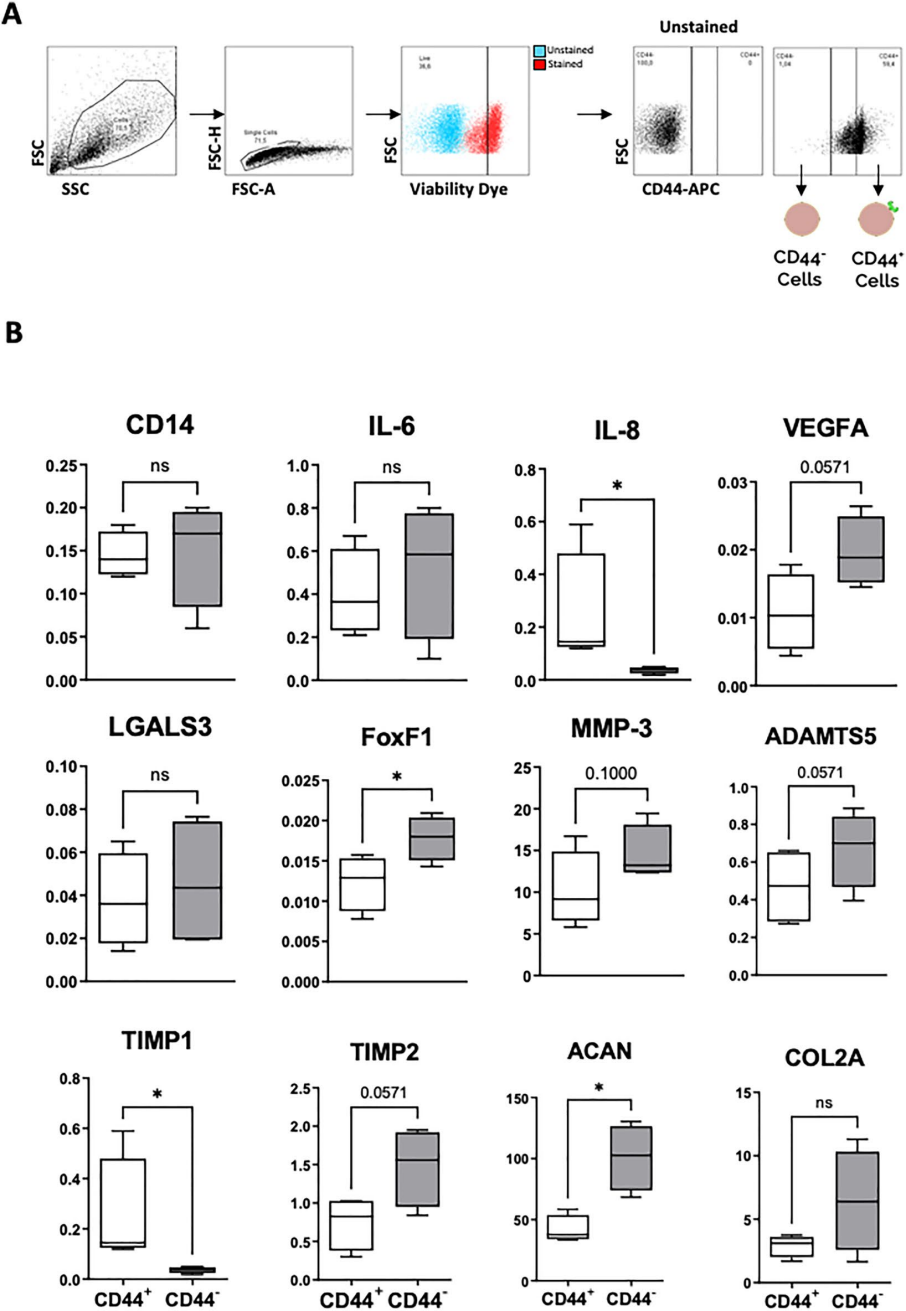
Gene expression profile of CD44^+^ versus CD44^-^ bovine NP cells. Fresh bovine NP cells were sorted for the expression of CD44 and analyzed for the gene expression of phenotypic markers. (**A**) Acquired cell sorting events were filtered based on: (i) size (FSC/SSC); (ii) symmetry for the removal of duplets (FSC-H/FSC-A); and (iii) viability (according to staining with viability dye). Filtered events were then separated into CD44^+^ and CD44^-^, according to the fluorescence intensity for the APC channel, and considering the non-specific fluorescence displayed by the unstained population. (**B**) Total mRNA isolated from the sorted cells was translated to cDNA and analyzed by qRT-PCR for the expression of: *CD14, LGALS3, FoxF1, IL-6, IL-8, VEGFA, ADAMTS5, MMP3, TIMP1, TIMP2, COL2A* and *ACAN* (*n* = 4). Results are presented as box and whiskers plots with representation of median, min and max values (statistical significance, *p < 0.05).

Aiming to extrapolate the functional identity of CD44^+^ cells within the IVD, freshly isolated NP cells were sorted based on CD44 expression and RNA was isolated from both CD44^+^ and CD44^-^ fractions (Fig. 1A). Gene expression of: an established monocyte/macrophage marker (*CD14),* NP phenotypic markers (*LGALS3*, *FoxF1, KRT8*, *KRT18*, *Pax1*, *TBXT*, *CD24* and *Tie2*), pro-inflammatory cytokines (*IL-6*, *IL-8*), pro-angiogenic factor (*VEGFA*), ECM proteases (*MMP3*, *TIMP1*, *TIMP2*, *ADAMTS5*) and ECM proteins (*COL2A1* and *ACAN*) was compared between the two sorted populations (Fig. 1B). Regarding *CD14 expression, a* monocyte/macrophage marker, no differences were observed between CD44^+^ and CD44^-^ cells. In what concerns NP lineage markers, CD44^+^ cells expressed significantly lower levels of *FOXF1* than their negative counterparts. The other NP lineage markers were not detected by qRT-PCR analysis. Among the expression of ECM proteins, matrix degrading enzymes and respective inhibitors, *ADAMTS5*, *TIMP2* (both *p* = 0.0571) and *ACAN* (*p* < 0,05) gene expression was significantly higher in CD44^-^ cells, compared with CD44^+^ cells, suggesting a higher involvement of those cells in the maintenance of ECM homeostasis. On the other hand, *TIMP1* was significantly more expressed by CD44^+^ cells (*p* < 0.05). In the context of the pro-inflammatory cascade, both CD44^+^ and CD44^-^ cell populations express similar levels of *IL-6*, while *IL-8 expression* was significantly increased in CD44^+^ cells (more than sixfold, *p* < 0.05). Lastly, the contribution of each cell fraction to the stimulation of degeneration-related neo-angiogenesis was hinted by the expression of *VEGFA*, which was found to be higher in CD44^-^ cells (*p* = 0.0571).

### CD44^+^ and CD44^***-***^ NP cells present a distinct proteomic profile

**Figure 2:**
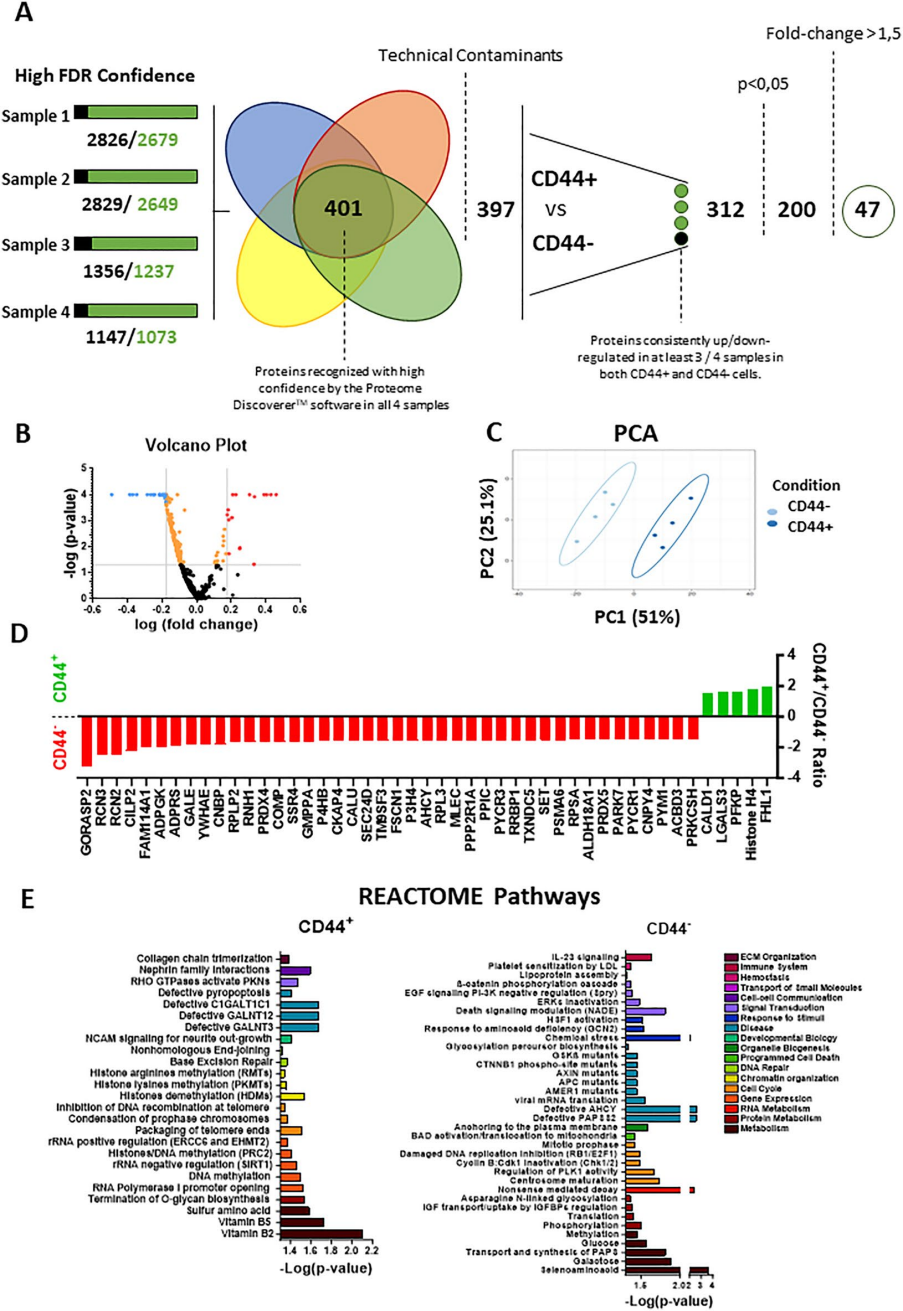
Proteomic analysis of CD44^+^ vs CD44^-^ bovine NP cells. Proteomic analysis of fresh NP cells sorted based on expression (or lack) of CD44. (**A**) Scheme of the protocol used to compare the proteomic profile of CD44^+^ vs CD44^-^ bovine NP cells. (**B**) Principal component analysis (PCA) results. (**C**) Volcano plot with threshold lines for *p*-value < 0.05 (Y axis) and CD44^+^/CD44^-^ fold-change >|1.5| (X axis). (**D**) proteins found significantly altered between CD44^+^ and CD44^-^ cells (fold-change >|1.5|, *p* < 0.05). (**E**) REACTOME software analysis of the proteins significantly up-regulated in CD44^+^ vs CD44^-^ bovine NP cells.

To distinguish the functional role of CD44^+^ vs CD44^-^ IVD cells, proteomic analysis was performed in sorted cell fractions to compare both cell populations (heatmaps presented in supplementary data, Fig. S1). A total of 397 proteins were detected with high confidence considering the 4 biological replicates (Fig. 2A). From the 397 proteins, 312 were proteins commonly identified in at least 3 out of 4 samples of each of the populations (CD44^+^ vs CD44^-^) (supplementary tables, Tables S1 and S2). From the 312 hits, 47 proteins were altered more than 1.5-fold when comparing CD44^+^ and CD44^-^ NP cells (Fig. 2B). A PCA analysis was performed, revealing a clear segregation between the two populations, i.e. distinct proteomic profiles (Fig. 2C). Among the differentially expressed candidates, 5 proteins were detected at significantly higher levels in CD44^+^ cells (Fig. 2D): (1) CALD1 (bovine non-muscle caldesmon), an actin- and myosin-binding protein implicated in the regulation of actomyosin cell interactions, that has been identified in single-cell RNA analysis of bovine IVD (interestingly associated to CD44 and LGALS3)^20^; (2) LGALS3 (galectin-3), a protein that plays an important role in cell–cell adhesion, cell–matrix interactions, macrophage activation, angiogenesis, metastasis, apoptosis, and that has been linked to increased IVD degeneration and ageing ^21^; (3) PFKP (Phosphofructokinase Platelet), an enzyme that catalyzes the phosphorylation of D-fructose 6-phosphate to fructose 1,6-bisphosphate by ATP, the first committing step of glycolysis, that has been reported to be dysregulated in IVD degeneration^22^; (4) Histone H4, one of the 5 main histone proteins involved in the structure of chromatin in eukaryotic cells, that has been identified as one of the molecules that could be used as a scoliosis biomarker^23^; and (5) FHL1 (Four-and-a-half LIM protein 1), a protein highly expressed in skeletal and cardiac muscles, which has been described to be down-regulated in chondrocytes from osteoarthritic patients^24^. Concerning CD44^-^ NP cells, the enrichment in 42 proteins (in opposition to 5 proteins enriched in CD44^+^ NP cells, Fig. 2D), and the lack of significant clustering by gene ontology (GO) analysis on Pearson correlation-defined hierarchical clusters, suggests that this population does not have a precise functional role.

An in-depth analysis of the pathways statistically affected by the significantly altered proteins (those that were 1.5 × higher either in CD44^+^/CD44^-^ fraction compared with the counterpart) was performed using the REACTOME software (Fig. 2E). This analysis demonstrated a significant enrichment of signaling pathways involved with Gene Expression, Chromatin Organization, DNA Repair, Developmental Biology, Cell–cell communication and ECM Organization only in CD44^+^ cells. For CD44^-^ NP cells, the masters signaling pathways affected were related to RNA Metabolism, Programmed Cell Death, Organelle Biogenesis, Response to Stimuli, Transport of Small Molecules, Hemostasis and Immune System. For those pathways commonly affected in both populations, a shift was observed. On the other hand, CD44^+^ NP cells appear to change their metabolic activity from a metabolism of selenoamino acids, glucose, galactose and transport/synthesis of PAPS (necessary to secure the successful production of proteoglycans^25–27^ to a metabolism of vitamins B2/B5 (riboflavin/pantothenate) and sulfur amino acids (necessary for the successful deposition of collagen^28–30^). In parallel, CD44^+^ NP cells significantly decrease their production of proteins involved with the biosynthesis of O-glycans and asparagine N-linked glycosylation, necessary to produce mucins, GAGs and collagen molecules^31,32^. Moreover, although both populations have a significant enrichment in upregulated proteins involved with the cell cycle, in CD44^+^ NP cells these proteins affect preferentially the regulation of rRNA and DNA/histones methylation whereas in CD44^-^ cells this preference falls upon the inhibition of DNA replication and the Cyclin B:Cdk1 complex and the regulation of PLK1 activity, centrosome maturation and mitotic prophase. Proteins found to be significantly upregulated in CD44^+^ cells were determined to be involved in mechanisms of avoidance of genome instability (packaging of telomere ends and the inhibition of DNA recombination at these structures) and, consequently, cell senescence protection^33–35^. Simultaneously, proteins implicated in mechanisms leading to genome instability (through the inhibition of damaged DNA replication)^36^, the activation of programmed cell death (due to BAD activation and or NADE modulation)^37,38^, the inhibition of the mitotic cycle (due to the inactivation of the Cyclin B:Cdk1 complex)^39^, and cell senescence induction (through the regulation of IGF uptake by IGFBPs)^40^ were significantly downregulated. In accordance, there was a decrease in the production of proteins involved with ERKs inactivation and negative regulation of EGF signaling, pathways reported to affect NP cell proliferation and viability^41–43^. Deregulated pathways were also detected for the two CD44 cell fractions. For CD44^+^ cells, these alterations were centered in the activity of C1GALT1C1, GALNT12, GALNT3, involved with protein glycosylation^44–46^, and during pyroptosis. In the case of CD44^-^ cells, the changes involve a wider range of altered proteins (GSKβ, CTNNB1, AXIN, APC, AMER1, AHCY and PAPSS2) and associated metabolic pathways (glycosylation biosynthesis and viral RNA translation). Lastly, with the acquired CD44 expression, NP cells shift their signaling transduction from β-catenin phosphorylation, ERKs inactivation and EGF/Death signaling to RHO GTPases signaling.

### IL-1β increases inflammatory markers and CD44 expression in NP cells

We also explored the effect of IL-1β on CD44 expression in IVD cells using a standardized pro-inflammatory/degenerative bovine NP organ culture model (needle puncture + IL-1β 10 ng/mL) previously established^17^.

**Figure 3:**
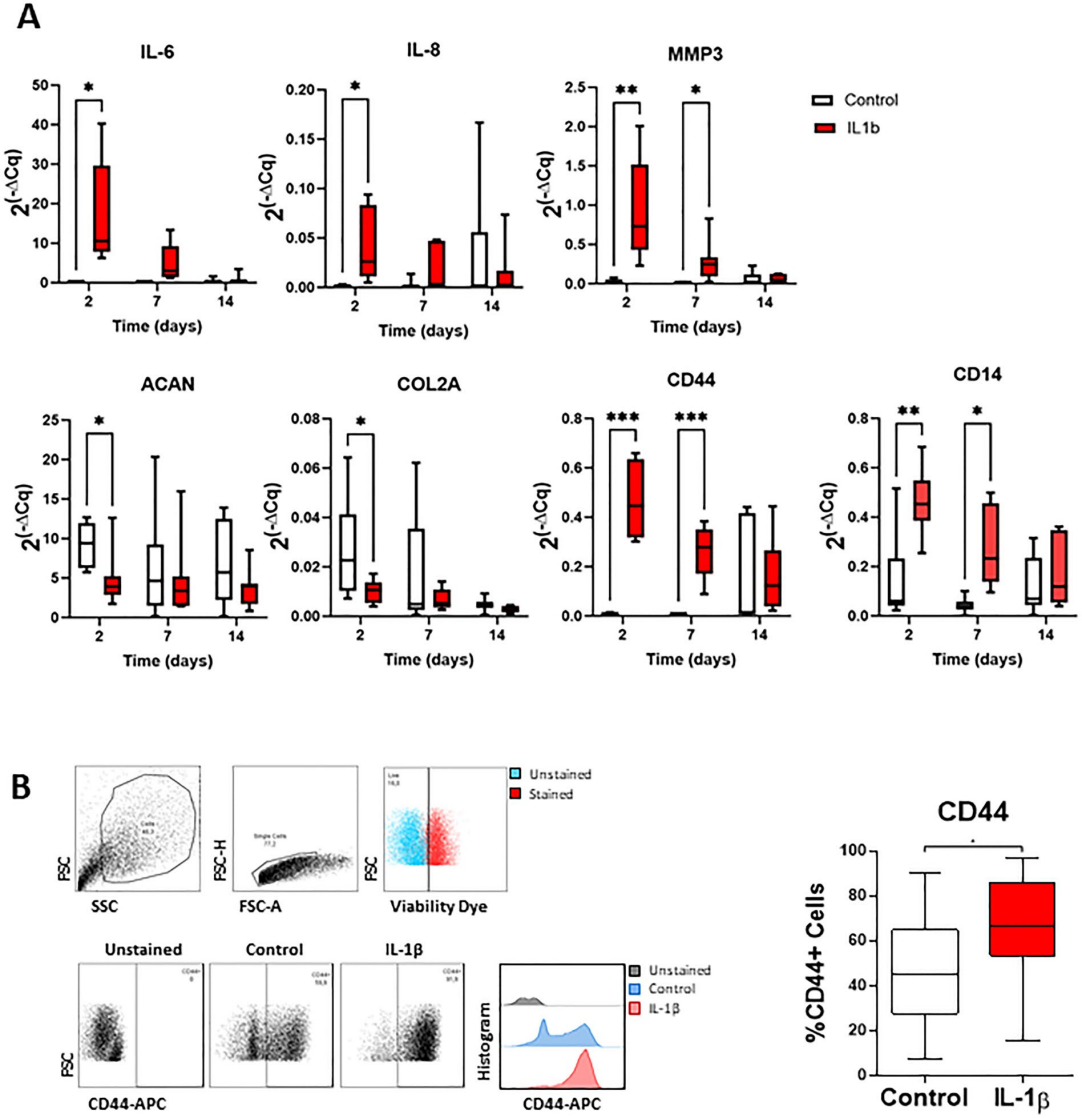
NP CD44 expression in response to a pro-inflammatory stimulus. CD44 expression was analyzed in bovine NPs stimulated by needle puncture and IL-1β. (**A**) IL-6, IL-8, MMP3, ACAN, COL2A, CD44 and CD14 gene expression were analyzed in days 2, 7 and 14 (*n* = 3–8). (**B**) CD44 cell surface quantification by flow cytometry (*n* = 22). Results are depicted as box and whiskers plots with representation of median, min and max. Statistical significance (*p < 0.05); **p < 0.01); ***p < 0.001).

First, gene expression of inflammatory players, MMPs and anabolic factors, as well as *CD14 and CD44* were evaluated in bovine NP under basal and pro-inflammatory conditions up to 14 days (Fig. 3A). Results show that all the inflammatory markers investigated (*IL-6, IL-8, CD14*, *CD44)* and *MMP3* were significantly upregulated in response to the degenerative stimulus, specially at day 2, in an acute phase, when compared with the unstimulated control. This up-regulation was decreasing over time, which was expected due to the lack of continuous IL-1β supplementation. In parallel, a significant down-regulation of ECM genes, *COL2A* and *ACAN*, was observed, confirming the effect of IL-1β in IVD anabolism.

To further confirm these observations, IVD tissue was digested and flow cytometry analysis of CD44 was also conducted in isolated NP cells (Fig. 3B), validating the gene expression data. A significative increase of CD44^+^ NP cells in response to the pro-inflammatory/degenerative stimulus was observed (from 48 ± 25% to 65 ± 23%, *p < 0.05).

**Figure 4:**
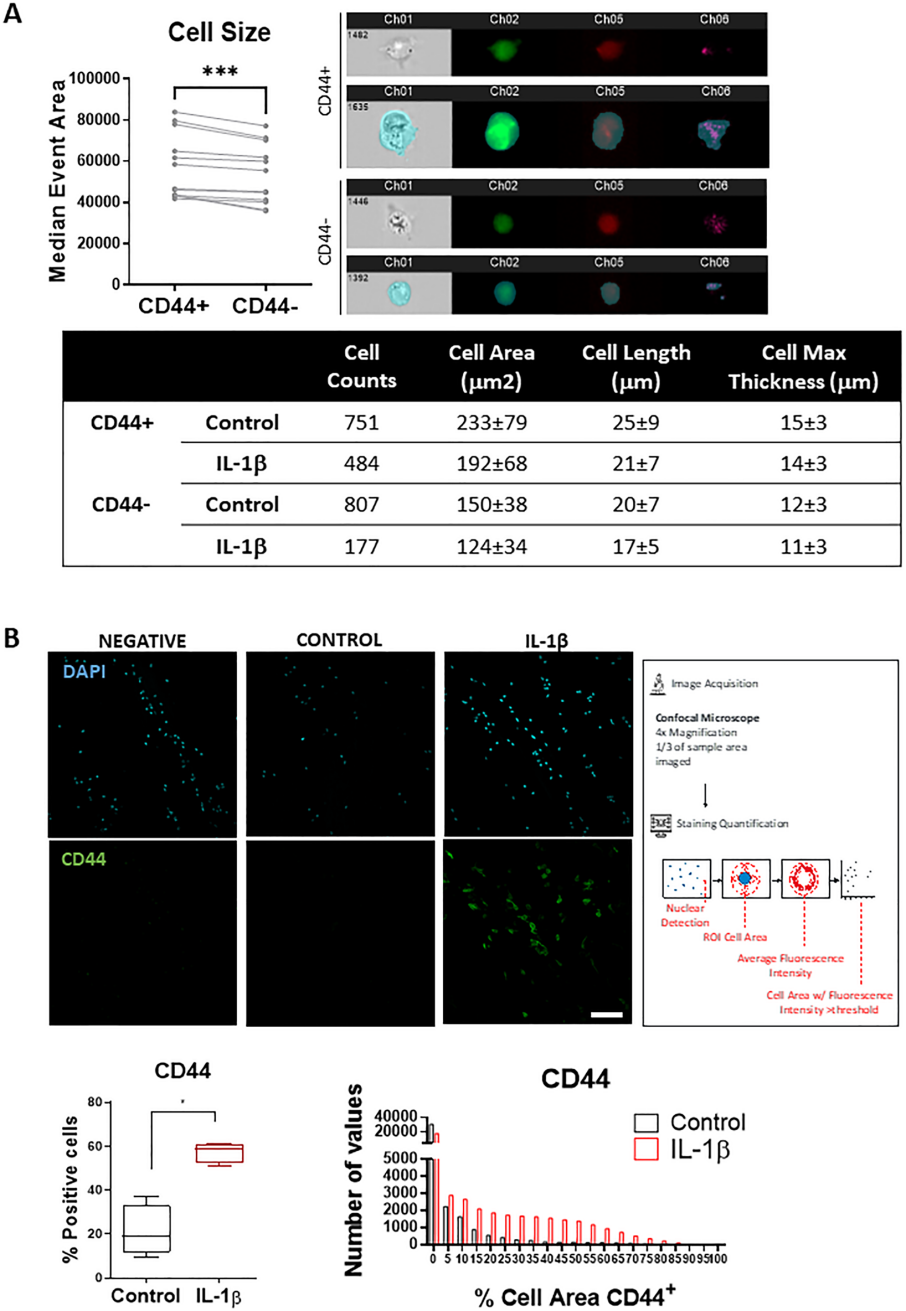
Morphological analysis of CD44 NP cells by imaging flow cytometry, conventional flow cytometry and immunofluorescence. (**A**) Morphological characterization of CD44^+^ vs CD44^-^ bovine NP cells through: (a) flow cytometry (difference in size assessed by backgating analysis). Results are presented as box and whiskers plots with representation of median, min, and max values (*n* = 12); (b) imaging flow cytometry of bovine NP cells stimulated with IL-1β. FITC-CD44 antibody (green, Ch02) and DRAQ5 nuclear staining (red, Ch05) were used to complement NP cell imaging (in brightfied, Ch01 and darkfield, Ch06). Results are presented in a table indicating the number of cells quantified (*n* = 1). (**B**) CD44 immunofluorescence staining after 2 days of culture. Representative images of sagittal sections of IVD explants (scale bar: 50 μm) Quantification of the percentage of cells positive for CD44 was performed according with the scheme presented. Results are presented as box and whiskers plots with representation of median, min, and max values (*n* = 4). Distribution of CD44^+^ cells according to percentage of the cell area stained for each marker is represented as an histogram for average value of *n* = 4.

**Figure 5:**
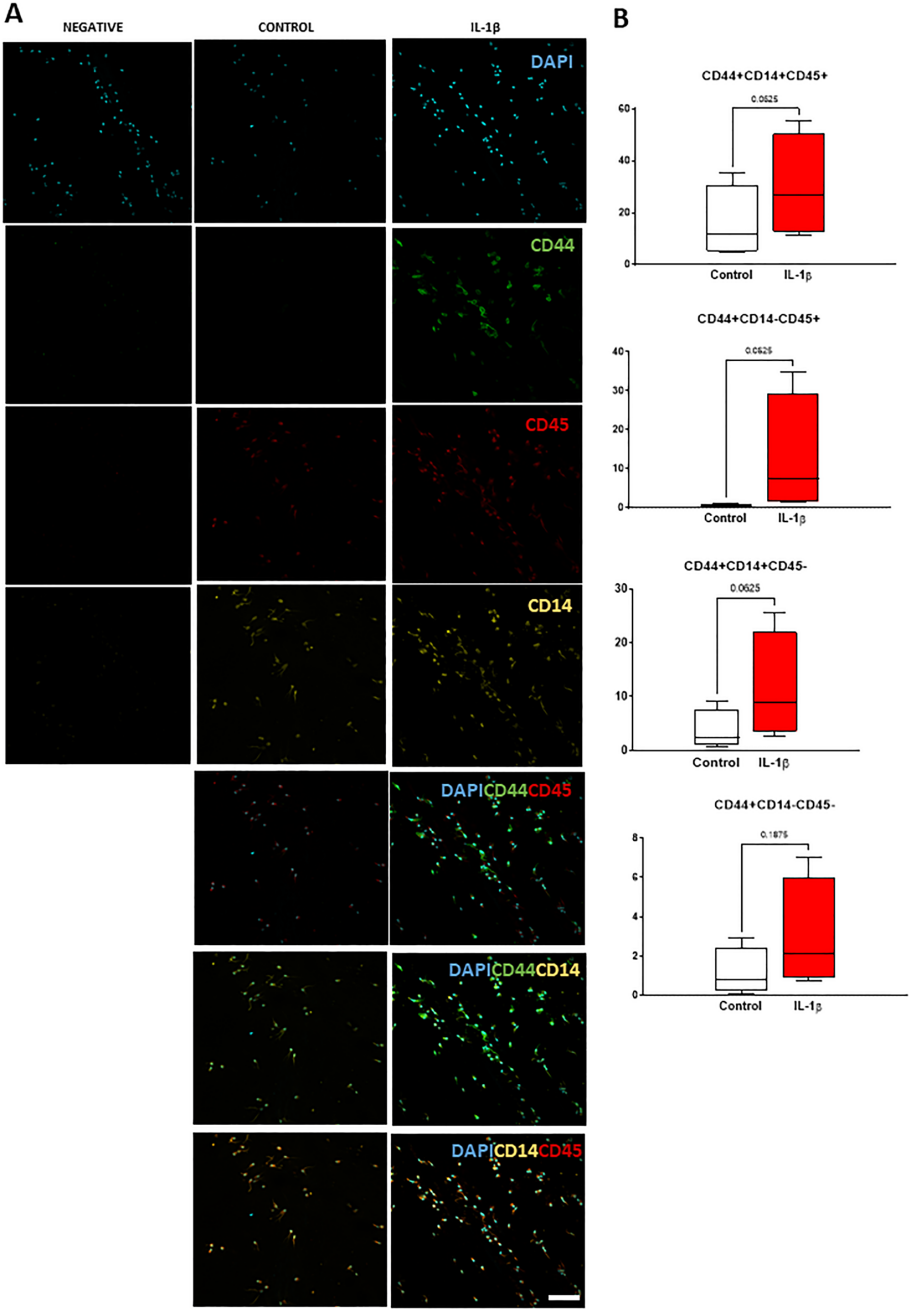
Co-expression analysis of CD44, CD45 and CD14 in NP cells in response to a pro-inflammatory/degenerative stimulus. (**A**) Representative images of sagittal sections of IVD explants with immunofluorescence staining for CD44, CD45 and CD14 in bovine NPs after 2 days in culture (scale bar: 50 μm). (**B**) Quantification of the percentage of cells simultaneously expressing CD44, CD45 and CD14 markers was performed, as previously described (*n* = 4). Results are presented as box and whiskers plots with representation of median, min and max. Statistical analysis was performed and p values indicated in the plots.

In addition, morphological analysis of CD44^+^ NP cells was also conducted. First, back gating analysis of flow cytometry analysis of CD44^+^ NP cells revealed a significant increase in mean cell area compared with their CD44^-^ counterpart (Fig. 5A). Then, imaging flow cytometry analysis was conducted on NP cells stimulated with IL-1b, revealing an increased cell size (area, length and thickness) in CD44^+^ compared to CD44^-^ NP cells, suggesting differences in morphology of both populations. However, the size differences seem to be reduced upon IL-1β stimulation (Fig. 4A).

Furthermore, we evaluated CD44 protein expression in the NP by immunofluorescence both in pro-inflammatory and basal conditions (Fig. 4B). Analysis of the CD44 tissue expression in bovine NP, at both cell cytoplasmic and membrane-bound forms (by using vs avoiding the membrane permeabilizer detergent (Tween-20) in the protocol) was performed (supplementary data, Fig. S2). The presence of the fluorescence signal for CD44 was then quantified by digital image processing and analysis (Fig. 4B). Results show that CD44^+^ NP cells significantly increased in IL-1β–treated IVDs compared to the control condition (*p < 0.05), confirming gene expression and flow cytometry data. A histogram to assess the distribution of NP cells in terms of percentage of CD44 stained area in both basal and pro-inflammatory conditions was performed. Histogram analysis demonstrates that most of the NP cells in basal conditions have low CD44 staining area which is increased upon IL-1β– stimulation (Fig. 4B).

### IL-1β increases the number of several CD44^+^ populations: CD44^+^CD14^+^CD45^+^, CD44^+^CD14^+^CD45^***-***^ and CD44^+^CD14^***-***^CD45^***-***^ NP cells

Having found a strong association between IL-1β stimulation and CD44 expression we next investigated whether CD44, CD14 (monocyte/macrophage marker) and CD45 (pan-hematopoietic marker), co-localized to identify the presence of IVD tissue resident macrophages (Fig. 5A). The percentage of cells co-expressing the possible combination of the three markers was assessed, resulting in 4 subpopulations CD44^+^CD14^+^CD45^+^, CD44^+^CD14^-^CD45^+^, CD44^+^CD14^+^CD45^-^ and CD44^+^CD14^-^CD45^-^ (Fig. 5B). The CD44^+^CD14^+^CD45^+^ NP cell subset (hematopoietic-derived monocyte/macrophage-like IVD cells) was the most frequent (15.8 ± 14.0%), followed by the CD44^+^CD14^+^CD45^-^ (3.7 ± 3.8%) (CD14-expressing NP cells, most probably similar to CD14-chondrocytes). CD14 expression has been described in human articular chondrocytes, but its role beyond its known immune functions remains to be highlighted ^47^. The other 2 cell subsets were present at low frequency in native tissue: CD44^+^CD14^-^CD45^-^ (1.2 ± 1.2%) (non CD14-NP cells) and CD44^+^CD14^-^CD45^+^ (0.6 ± 0.4%) (hematopoietic-derived myeloid-non-monocytic NP cells). With IL-1β stimuli and accompanying the CD44 increase in the NP already described, 3 (out of 4) CD44^+^ cell subsets increased, although did not reach a statistical significance (*p* = 0.0625): CD44^+^CD14^+^CD45^+^ (twofold increase), CD44^+^CD14^+^CD45^-^ (threefold increase) CD44^+^CD14^-^CD45^-^ (24-fold increase) suggesting that CD44-expressing NP cell subpopulations might play an important role in inflammatory cascade associated with IVD degeneration.

## Discussion

In this study, we have investigated the CD44 response to IL-1β in IVD degeneration.

CD44 refers to a family of widely expressed cell surface glycoproteins primarily involved in cell-ECM adhesion mechanisms^48^. CD44 has been consistently identified as a marker of NP cells across species^5^, but it is also expressed by macrophages, having an important role in monocyte differentiation^49^. In human IVDs, CD44 has been described as highly expressed (> 90%)^50^. CD44 main ligand is hyaluronic acid (HA)^6^, one of the most abundant glycosaminoglycans (GAGs) in the NP^7^. CD44 was shown to mediate the catabolic effects observed when treating chondrocytes with IL-1β^51^. Here we reported an increase of CD44^+^ NP cells in response to the pro-inflammatory/degenerative stimulus. If we improve our knowledge on this cell population in an inflammatory context, CD44 NP cells could be used as a therapeutic target.

Gene expression analysis of CD44^+^ and CD44^-^ NP cells revealed that both populations expressed similar levels of *CD14* and *LGALS3*, something that was confirmed at the protein level for CD14 but was challenged by the detection of a significant increase in the production of Galectin in CD44^+^ cells. This may be explained by the fact that not only *LGALS3* but also Gal-1 are expressed in the IVD^21^. Gal-1 seems to be exclusively involved with the induction of pro-inflammatory/degenerative players (such as MMPs, IL-6 and IL-8), while L*GALS3* seems to be associated with age^21^. Given that in this work young bovine IVD donors were used, while IVD ageing and IVD degeneration may represent distinct concepts, it is reasonable to hypothesize that this increase may be specifically due to Gal-1.

On the other hand, CD44^+^ NP cells expressed significantly less *FoxF1* than CD44^-^ cells. *FOXF1* is a transcription factor highly linked to the regulation of embryogenesis, specifically during mesodermal differentiation^52^, being necessary for the vasculature formation during this phase^53^. Its expression has been reported as a solid NP cell marker^54,55^, and its levels shown to decrease with increased degeneration^55^, in accordance with our results. Additionally, *COL2A* and *ACAN* transcription levels can also be used as phenotypic markers of NP cells. In this work, CD44 expression was shown to be accompanied by a downregulation of both genes, as observed in degenerated IVDs^56^. Regarding the expression of other degeneration intermediates, an increase in *IL-8* and matrix degrading enzymes inhibitor *TIMP1* and a decrease in most genes related with the ECM maintenance (*TIMP2*, *ADAMTS5*, *ACAN*, *COL2A*, and *MMP3*) were observed in CD44^+^ cells. As such, expression of CD44 seems to be linked to the mediation of the inflammatory cascade through IL-8 signaling and ECM catabolism regulation through TIMP1.

By proteomic analysis of CD44^+^ vs CD44^-^ NP cells, we observed that CD44^+^ NP cells present an increased production of proteins necessary for the successful deposition of collagen, and a decreased production of proteins involved in the metabolism of proteoglycans. This indicates a link between CD44 expression and collagen synthesis. More specifically, CD44^+^ NP cells produce more collagen type VI and less collagen type II than their negative counterparts. Collagen type VI is very abundant in the NP (20% of total collagen) as part of the cells’ pericellular matrix^57^ and has been found increased in areas of minor to advanced degeneration^58^, due to deregulation of ECM production during IVD degeneration. On the other hand, its expression has also been linked to peripheral nerve regeneration in mice^59^, which correlates well with the observation that expression of CD44 upregulates factors involved with NCAM signaling, that functions as an attractive cue for neurite outgrowth^60^. In the context of degenerated IVD, and in the absence of increased CD44 expression, this may entail an imbalance in neuronal guidance cues that could promote invasion of the tissue by nerves, a hallmark of the degenerative process directly linked with the onset of LBP^61^.

CD44^+^ NP cells also showed a decrease of the defective activity of several members of the Wnt/β-catenin destruction complex (e.g. AMER1, APC, AXIN, CTNNB1 and GSKβ). This unrestrained activation of the Wnt pathway, may lead to uncontrolled cell proliferation^62,63^, and impaired NP cell differentiation^64^. In fact, high levels of β-catenin paired with decreased Wnt activity were observed in patients with IVD degeneration^65^. In addition, an increased production of proteins (DNA and histones methylation/demethylation mechanisms^66^ and increased activity of EHMT2 and ERCC6^67^), has been previously shown to be linked to IVD degeneration^68^. Such results implicate CD44 as an important regulator of IVD degeneration.

Moreover, we dissected the response of CD44^+^ NP cells to IL-1β, demonstrating that this cell subset increases in a pro-inflammatory microenvironment, together with other pro-inflammatory mediators. Moreover, CD44 has been reported to initiate cytoskeletal rearrangement through the modulation of RHO GTPases activity in other cells, including astrocytes^69^, T cells^70^ and neurons^71^. In these cases, the reorganization of the actin cytoskeleton led to either a general increase in cell size, in cell spreading or in dendritic spine elongation, respectively, all concordant with the effects herein observed on NP cells. Posterior analysis of the CD44^+^ versus CD44^-^ NP cells’ proteome also confirmed the significantly increased production of proteins linked to the activation of RHO GTPases with the expression of CD44.

Furthermore, we have investigated the co-expression of CD44 with CD14 and CD45, intending to deepen the knowledge on the presence of tissue resident macrophages in NP. For that we have used a standardized ex vivo model of NP inflammation/degeneration, that our group was pioneer in establishing^17^. In this model, we have identified the presence of a cell subset of CD44^+^CD45^+^CD14^+^ NP cells, suggesting the presence of hematopoietic-derived monocyte/macrophage-like cells in native IVD. Another hematopoietic-derived population myeloid-derived non-monocytic (CD44^+^CD14^-^CD45^+^) was observed in very low frequency in native IVD, but increasing substantially with IL-1β. Moreover, other NP cell subsets without hematopoietic origin expressing CD44, as CD44^+^CD14^+^CD45^-^, revealed to be present in native IVD. Although CD45 is widely known as a pan-marker of hematopoietic cells^72^, more recently, its expression has also been detected in mesenchymal stromal cells (MSCs)^73^. This opens the possibility for its expression in other cells of mesenchymal origin. CD45 main function is mediated by its intracellular segment’s phosphatase activity, whose major targets are the Src-family kinases (SFK)^74^, which phosphorylation has been involved in the central sensitization and radicular pain from lumbar discs’ herniation^75^. Interestingly, SFKs’ phosphorylation has also been associated with *COL2A1* and *ACAN* expression in NP cells under periodic mechanical stress^76^. In our study CD45 increase is linked to the expression of CD44. Other studies have shown that the expression of high levels of CD45 linked to an increased gain of an activated/phagocytic phenotype^77^. Likewise, although CD14 has been reported mostly as a monocyte/macrophage lineage marker, it has also been detected in non-myeloid cells, including endothelial cells, epithelial cells, smooth muscle cells and fibroblasts^78^. Its main function is to recognize patterns of receptors expression on the surface of cells and to respond to signalling via LPS, leading to the intense production of pro-inflammatory cytokines^78^. In fact, comparative analysis between CD14^+^/CD14^-^ human IVD cells showed that CD14^+^ cells produced significantly higher levels of TNF-α, IL-1β, IL-6 and NGF^79^. Moreover, TNF-α-stimulation of CD14^-^ IVD cells up-regulated NGF and CGRP production. This strongly indicates that CD14^+^ cells may be responsible for the strong inflammatory cascade originated in the degenerated IVD.

An increasing number of reports suggests the presence of resident phagocytic cells^80^ and cells of myeloid origin^8^ in the native, non-herniated, IVD. Nevertheless, this is still a controversial issue in the field. Some authors have reported that these cells lack the expression of certain myeloid markers, such as CD68^81^, while others have confirmed the presence of other myeloid markers (as F4/80^+^CD45^+^CD11b^+^) in intact discs^8^, indicating the existence of tissue-resident macrophages. Moreover, a much more heterogeneous cell compartment in the IVD has been increasingly reported, with the existence of different populations of immune-like or myeloid-derived cells expressing markers such as CD11b concomitantly expressed with the NP progenitor marker CD24^82^. This observation is in line with the presence of a high percentage of myeloid cells in bovine IVD^83^. Moreover, the expression of CD44, CD14 and CD45 has also been described in the aneural and avascular human lens in the context of responders to injury^84^, suggesting that in IVD these cells might also play similar role. Nevertheless, the knowledge on myeloid-derived cells in the IVD is still on its infancy. Future work further elucidating the function and activity of these IVD cell subsets in the context of the degenerative cascade could provide important insights on new therapeutic targets.

## Conclusions

This work explored the CD44 dynamics triggered by IL-1β stimulation of NP. CD44^+^ NP cells were suggested to favor collagen type VI production over collagen type II and proteoglycans, leading to a shift in ECM production. In the NP, CD44 expression appears to lead to a heightened cytoprotective metabolic state, avoiding extensive cell death. Based on the multiple pathways affected by CD44, its expression in NP cells appears to induce a cell differentiation process that leads to an increased cytoprotective activity, a shift on ECM profile and a stimulation of neuro-permissive factors. Moreover, we demonstrated that CD44 is stimulated by IL-1β, with alterations in cell morphology, and that bovine NP cells co-express CD44 with monocyte/macrophage lineage-tracing markers, CD45 and CD14, that increase with the presence of an inflammatory stimulus, thus potentially playing an important role in the IVD pro-inflammatory/catabolic cascade. Overall, this study opens insights into novel therapeutic targets in IVD degeneration.

## Supplementary Information

Supplementary Information.

## Data Availability

The mass spectrometry proteomics data have been deposited to the ProteomeXchange Consortium via the PRIDE partner repository with the dataset identifier PXD048373.
