# Author Correction: Dynamics of CD44^+^ bovine nucleus pulposus cells with inflammation

**DOI:** 10.1038/s41598-025-90561-8

**Published:** 2025-03-04

**Authors:** J. R. Ferreira, J. Caldeira, M. Sousa, M. A. Barbosa, M. Lamghari, G. Almeida-Porada, R. M. Gonçalves

**Affiliations:** 1 I3SI3—Instituto de Investigação e Inovação em Saúde, Universidade Do Porto, Porto, Portugal; 2 INEB—Instituto de Engenharia Biomédica, Universidade Do Porto, Porto, Portugal; 3 Instituto de Ciências Biomédicas Abel Salazar, Universidade Do Porto, Porto, Portugal; 4 WFIRM—Wake Forest Institute for Regenerative Medicine, Winston-Salem, NC USA; 5 Present Address: Cell & Gene Therapy Safety, Clinical Pharmacology & Safety Science, R&D, AstraZeneca, Molndal, Sweden

Correction to: *Scientific Reports* 10.1038/s41598-024-59504-7, published online 21 April 2024

The Funding section in the original version of this Article was incomplete.

“This work has been performed with funding from the Portuguese Foundation for Science and Technology (FCT) (UIDB/04293/2020) and AOS-Startup-23-022: Stop & Rewind: CRISPR-mediated fetal gene activation for IVD regeneration.”

Now reads:

“Project No AOS Startup 23-022 was supported by AO Spine. AO Spine is a clinical division of the AO Foundation—an independent medically-guided not-for-profit organization based in Davos, Switzerland. This work has been also performed with funding from the Portuguese Foundation for Science and Technology (FCT) (UIDB/04293/2020).”

The original Article has been corrected.

