# Supplementary material for: Dynamics of CD44+ bovine nucleus pulposus cells with inflammation
Source: Sci Rep. 2024 Apr 21;14:9156. doi: 10.1038/s41598-024-59504-7 (PMC11033282; doi:10.1038/s41598-024-59504-7)
Supplement: Supplementary file 1 — Supplementary Information. [file 41598_2024_59504_MOESM1_ESM.docx]

**Supplementary Material**

**Supplementary Figure 1**

**
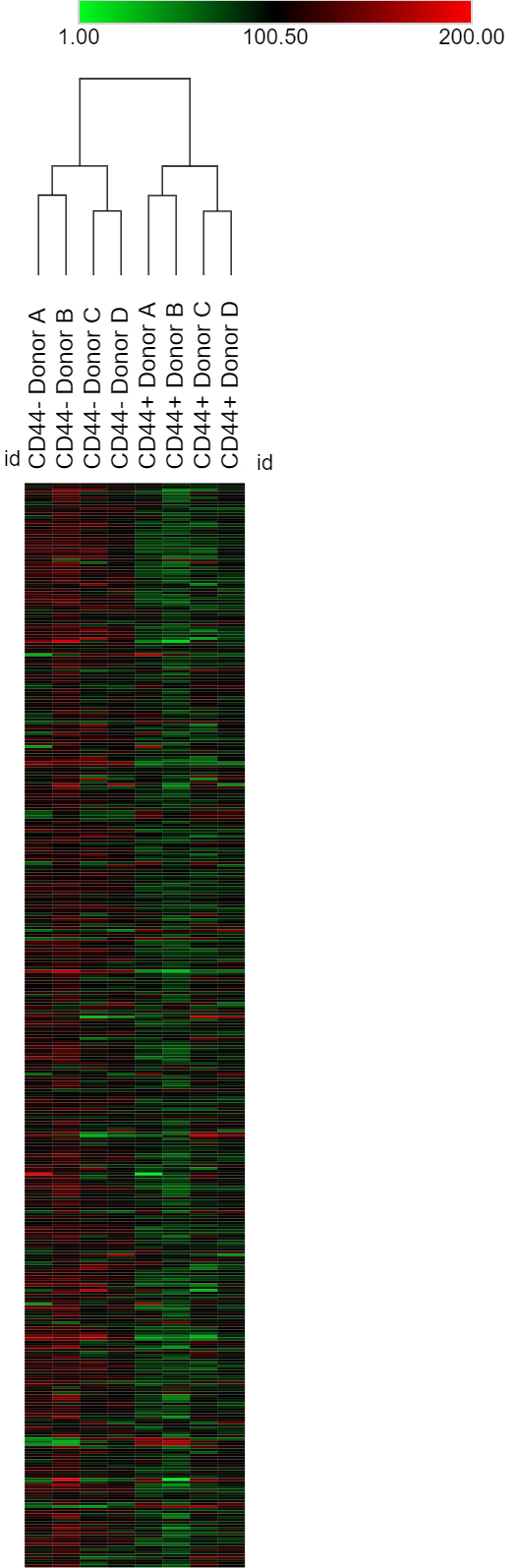
Figure S1. Heatmap of the proteomic profile of CD44+ vs CD44- bovine NP cells for the 8 replicates analyzed.**

Heatmap of proteins identified simultaneously in all four donors of the fresh bovine NP cells analyzed, made with Morpheus. Protein relative concentration are displayed with colours ranging from green (minimum limit: 1) to red (maximum limit: 200) as shown in the key. Columns are clustered using the one minus Pearson correlation metric and average linkage.

**Figure S2. Comparison of CD44/CD14/CD45 in permeabilized vs non-permeabilized NP cells**

Immunofluorescence staining for CD44, CD45 and CD14 in the degenerative NP tissue with permeabilization (incubation/washing steps with 0,05% Tween-20) vs non-permeabilization (incubation/washing steps without 0,05% Tween-20) conditions. Representative images of sagittal sections of NP explants stained for CD44, CD45 and CD14 (scale bar: 50 μm)

**
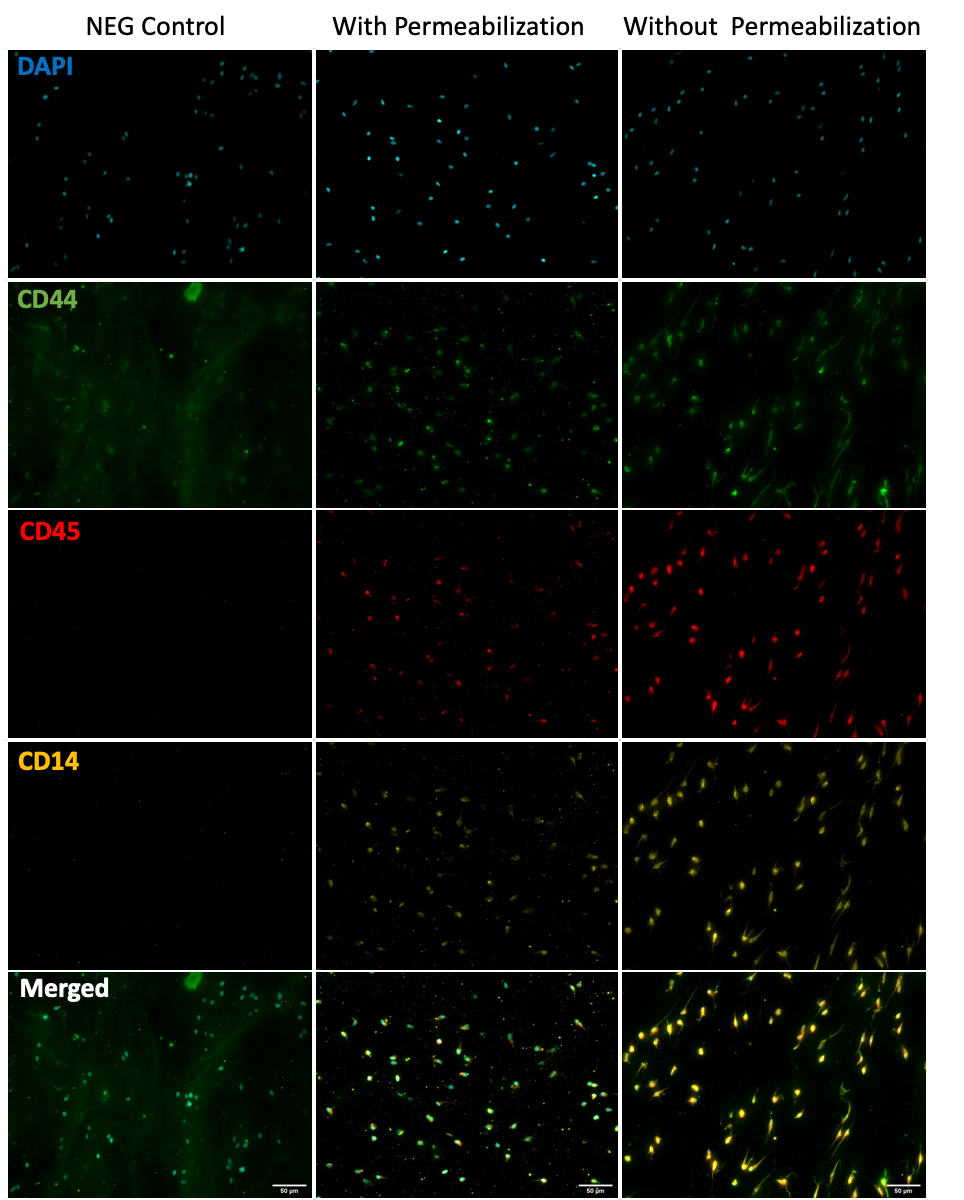
**

**Table S1. Genes sequence**

| **Function** | **Gene** | **Sequence** | **Amplicon Length (bp)** | **NCBI Ref.** |
| --- | --- | --- | --- | --- |
| **House keeping** | ***bGAPDH*** | Fw: 5’-GAA GGT GAA GGT CGG AGT C-3’ | 224 | J04038 |
|  |  | Rv: 5’-GAA GAT GGT GAT GGG ATT TC-3' |  |  |
| **HA receptor** | ***bCD44*** | Fw: 5'-CTG GGG ACT CTG CCT CGT-3' | 120 | NC_037342.1 |
|  |  | Rv: 5'-CAC GTG GAA TAC ACC TGC ATA-3' |  |  |
| **Monocyte/ macrophage lineage** | ***bCD14*** | Fw: 5'-CCG ACA ACC AGA GAG AGA GG-3' | 180 | NC_037334.1 |
|  |  | Rv: 5'-TCG CAG GGT TCT GTT GTG T-3' |  |  |
| **Hematopoietic lineage** | ***bCD45*** | Fw: 5'-TGG CTT AAA CTT CTG GCA TTC-3' | 153 | NC_037343.1 |
|  |  | Rv: 5'-TGA GTA GAA GGT TGG GTA GAG TTT C-3' |  |  |
| **NP Phenotype** | ***bFoxF1*** | Fw: 5'-ACA TC ACA ACA CCC CTG CT-3' | 161 | NC_037345.1 |
|  |  | Rv: 5'-GGT GAC CTG CTG GTG GTA GT-3' |  |  |
|  | ***bLGalS3*** | Fw: 5'-CCA ACA GCA CCT GCT TAT CC-3' | 227 | NC_037337.1 |
|  |  | Rv: 5'-CTG TTC GCA TTC GGC TTT AC-3' |  |  |
|  | **bKRT8** | Fw: 5'-GTC AAC GCC CAG TAT GAG GA-3' | 157 | NC_037332.1 |
|  |  | Rv: 5'-TGA TGT TCC GGT TCA TCT CA-3' |  |  |
|  | **bKRT18** | Fw: 5'-AGC CTC GAT CTC AGT CTC CA-3' | 193 | NC_037332.1 |
|  |  | Rv: 5'-TCG AAA TTC TGT GGA CAA CG-3' |  |  |
|  | **bPax1** | Fw: 5'-CCT GCC TTA GAC ACG GAC AT-3' | 248 | NC_037340.1 |
|  |  | Rv: 5'-TTG GGA TGC TTG AAG GTC AC-3' |  |  |
|  | **bBrachyury** | Fw: 5'-GAT GTG GAT TCG AGG CTC AT-3' | 209 | NC_037336.1 |
|  |  | Rv: 5'-AGT ACG TGA ATG GGG AGT GG-3' |  |  |
|  | **bCD24** | Fw: 5'-ACG AGG AGC TCC AAA AAC AA-3' | 231 | NC_037336.1 |
|  |  | Rv: 5'-GAT TGC AGG GTG CCG AAG-3' |  |  |
|  | **bTie2** | Fw: 5'-GGT CTT CAT TCT TGC CCT GA-3' | 206 | NC_037335.1 |
|  |  | Rv: 5'-AGC CAG AGT CAA CAC CAA GG-3' |  |  |
| **Angiogenesis** | ***bVEGFA*** | Fw: 5'-GAT GTC TAC CAG CGC AGC TT-3' | 219 | NC_037350.1 |
|  |  | Rv: 5'-TAT GTG CTG GCT TTG GTG AG-3' |  |  |
| **Inflammation** | ***bIL-6*** | Fw: 5’-AGG AGA CTT GCC TGG TGA AA-3’ | 180 | NM_000600 |
|  |  | Rv: 5’-CAG GGG TGG TTA TTG CAT CT-3’ |  |  |
|  | ***bIL-8*** | Fw: 5’-GTG CAG TTT TGC CAA GGA GT-3’ | 196 | NM_000584 |
|  |  | Rv: 5’-CTC TGC ACC CAG TTT TCC TT-3’ |  |  |
| **ECM proteases** | ***bMMP3*** | Fw: 5’-GGA GAT GCC CAC TTT GAT GAT-3’ | 187 | NM_002422 |
|  |  | Rv: 5’-CAT CTT GAG ACA GGC GGA AC-3’ |  |  |
|  | ***bADAMTS5*** | Fw: 5’-CAC CTC AGC CAC CAT CAC AG-3’ | 152 | NM_001166515.1 |
|  |  | Rv: 5’-AGT ACT CTG GCC CGA AGG TC-3´ |  |  |
|  | ***bTIMP1*** | Fw: 5’-GAC CGC AGA AGT CAA TGA AA-3’ | 176 | NM_174471.3 |
|  |  | Rv: 5’-GAA ACT CCT CAC TGC GGT TC-3’ |  |  |
|  | ***bTIMP2*** | Fw: 5´-AGG GCC AAA GCA GTC AAT AA-3’ | 150 | NC_037346.1 |
|  |  | Rv: 5’-AGA GGA GGG GGC TGT GTA GA-3’ |  |  |
| **ECM proteins** | ***bCOL2A*** | Fw: 5’-CGC ACC TGC AGA GAC CTG AA-3’ | 162 | XM_056481 |
|  |  | Rv: 5´-TCT TCT TGG GAA CGT TTG CTG G-3’ |  |  |
|  | ***bACAN*** | Fw: 5’-TCT GTA ACC CAG GCT CCA AC-3’ | 199 | XM_007701 |
|  |  | Rv: 5’-CTG GCA AAA TCC CCA CTA AA-3’ |  |  |

**Table S2. Table of proteins with statistically significant changes between CD44+ and CD44- cells (p<0,05) with a CD44+/CD44- ratio < 1,5.**

| **Uniprot ID** | **Name** | **CD44+/ CD44-** | **P value** |
| --- | --- | --- | --- |
| P48616 | Vimentin | 1,474 | 0,0184 |
| E1BB91 | Collagen type VI alpha 3 chain | 1,447 | 0,0021 |
| E1BI98 | Collagen type VI alpha 1 chain | 1,431 | 0,0059 |
| G5E631 | RNA helicase | 1,423 | 0,0039 |
| Q32LC7 | Ras GTPase-activating protein-binding protein 1 | 1,420 | 0,0090 |
| F6QQ60 | Tropomyosin 4 | 1,420 | 0,0346 |
| P04272 | Annexin A2 | 1,320 | 0,0164 |
| A0A3Q1LVC7 | Ezrin | 1,317 | 0,0346 |
| F1N169 | Filamin A | 1,305 | 0,0422 |
| F1MQ37 | Myosin heavy chain 9 | 1,296 | 0,0218 |
| P46193 | Annexin A1 | 1,278 | 0,0357 |
| Q0VBZ9 | MARCKS-related protein | 1,268 | 0,0408 |
| P02638 | Protein S100-B | 0,850 | 0,0004 |
| A0A3Q1LI03 | Peptidylprolyl isomerase | 0,841 | 0,0382 |
| Q3SYZ4 | Aspartate--tRNA ligase, cytoplasmic | 0,814 | 0,0485 |
| P61356 | 60S ribosomal protein L27 | 0,810 | 0,0436 |
| P41541 | General vesicular transport factor p115 | 0,809 | 0,0365 |
| F1MWE0 | Proteasome 26S subunit, ATPase 3 | 0,809 | 0,0413 |
| A2VDN7 | Heterogeneous nuclear ribonucleoprotein U | 0,809 | 0,0171 |
| Q32KL2 | Proteasome subunit beta type-5 | 0,808 | 0,0145 |
| Q3ZCC9 | Protein SEC13 homolog | 0,807 | 0,0193 |
| A6QP36 | LMAN2 protein | 0,807 | 0,0417 |
| P81623 | Endoplasmic reticulum resident protein 29 | 0,806 | 0,0338 |
| A6QLL8 | Fructose-bisphosphate aldolase | 0,804 | 0,0450 |
| A2VDL8 | Protein transport protein Sec23A | 0,804 | 0,0436 |
| Q2KHU8 | Eukaryotic translation initiation factor 2 subunit 3 | 0,803 | 0,0263 |
| A0A3S5ZPB0 | Fructose-bisphosphate aldolase | 0,803 | 0,0298 |
| A1A4J1 | ATP-dependent 6-phosphofructokinase, liver type | 0,802 | 0,0080 |
| P19858 | L-lactate dehydrogenase A chain | 0,802 | 0,0288 |
| E1B7U1 | Mannosyl-oligosaccharide glucosidase | 0,800 | 0,0316 |
| P13696 | Phosphatidylethanolamine-binding protein 1 | 0,800 | 0,0228 |
| A0JN39 | Coatomer subunit beta | 0,796 | 0,0288 |
| A0A3Q1MAU7 | S-formylglutathione hydrolase | 0,795 | 0,0100 |
| A0A452DJG7 | Dolichyl-diphosphooligosaccharide--protein glycosyltransferase subunit 2 | 0,795 | 0,0200 |
| A5PJR4 | Adenylosuccinate synthetase isozyme 1 | 0,794 | 0,0295 |
| P13213 | SPARC | 0,793 | 0,0027 |
| A0A452DIH7 | Tubulin alpha chain | 0,793 | 0,0298 |
| Q3T057 | 60S ribosomal protein L23 | 0,793 | 0,0223 |
| Q3SZ20 | Serine hydroxymethyltransferase, mitochondrial | 0,792 | 0,0350 |
| Q2KJH6 | Serpin H1 | 0,791 | 0,0330 |
| Q3B7M5 | LIM and SH3 domain protein 1 | 0,790 | 0,0278 |
| F6QH94 | Protein disulfide isomerase family A member 6 | 0,789 | 0,0260 |
| Q3SX08 | EMD protein | 0,788 | 0,0220 |
| A6QLT9 | Alanine--tRNA ligase | 0,787 | 0,0251 |
| Q3T169 | 40S ribosomal protein S3 | 0,786 | 0,0223 |
| A2VE53 | Inhibitor of nuclear factor kappa-B kinase-interacting protein | 0,785 | 0,0167 |
| A0A3Q1MQS3 | 14-3-3 protein theta | 0,784 | 0,0157 |
| Q9BGI3 | Peroxiredoxin-2 | 0,784 | 0,0101 |
| A0A3Q1MQ34 | Filamin B | 0,781 | 0,0220 |
| Q5E9A3 | Poly(rC)-binding protein 1 | 0,781 | 0,0210 |
| Q56JX8 | 40S ribosomal protein S13 | 0,780 | 0,0195 |
| Q0VCX2 | Endoplasmic reticulum chaperone BiP | 0,779 | 0,0223 |
| A0A3Q1M6K6 | Aconitate hydratase, mitochondrial | 0,778 | 0,0195 |
| A0A3S5ZP98 | Alpha-aminoadipic semialdehyde dehydrogenase | 0,777 | 0,0133 |
| F1MD34 | Niban apoptosis regulator 2 | 0,777 | 0,0065 |
| A7Z066 | Calnexin | 0,777 | 0,0220 |
| A6H7G2 | Drebrin-like protein | 0,777 | 0,0184 |
| E1BBC6 | Procollagen-proline 3-dioxygenase | 0,775 | 0,0210 |
| P61284 | 60S ribosomal protein L12 | 0,773 | 0,0083 |
| Q3T0J0 | 3'-phosphoadenosine-5'-phosphosulfate synthase | 0,773 | 0,0115 |
| Q3T0P6 | Phosphoglycerate kinase 1 | 0,773 | 0,0135 |
| Q2KJI2 | Dolichyl-diphosphooligosaccharide--protein glycosyltransferase subunit STT3A | 0,772 | 0,0184 |
| A0A3Q1LYJ9 | Signal sequence receptor subunit alpha | 0,772 | 0,0162 |
| Q32PA9 | Peptidyl-prolyl cis-trans isomerase FKBP2 | 0,772 | 0,0001 |
| A0A3Q1M0U3 | Sorbin and SH3 domain containing 3 | 0,770 | 0,0123 |
| F1MZY2 | Glutamine--fructose-6-phosphate transaminase (isomerizing) | 0,766 | 0,0147 |
| F1MLG1 | Signal recognition particle receptor subunit beta | 0,766 | 0,0087 |
| F1MK30 | 60S ribosomal protein L13 | 0,765 | 0,0138 |
| G5E5C8 | Transaldolase | 0,765 | 0,0140 |
| A1L5A6 | Proteasomal ubiquitin receptor ADRM1 | 0,764 | 0,0118 |
| P17248 | Tryptophan--tRNA ligase, cytoplasmic | 0,764 | 0,0136 |
| F1MB08 | 2-phospho-D-glycerate hydro-lyase | 0,763 | 0,0108 |
| Q58DT1 | 60S ribosomal protein L7 | 0,763 | 0,0091 |
| P02459 | Collagen alpha-1(II) chain | 0,763 | 0,0143 |
| A7Z014 | TKT protein | 0,759 | 0,0123 |
| Q3SZ90 | 60S ribosomal protein L13a | 0,759 | 0,0058 |
| A0A493UA87 | Aldo-keto reductase family 1 member B1 | 0,756 | 0,0063 |
| G3N2L2 | Reticulocalbin 1 | 0,752 | 0,0087 |
| A7MB83 | Procollagen-lysine 5-dioxygenase | 0,752 | 0,0075 |
| A6H709 | HSPC321 protein | 0,751 | 0,0068 |
| E1BFV0 | Karyopherin subunit beta 1 | 0,748 | 0,0084 |
| E1BLV6 | Valyl-tRNA synthetase | 0,746 | 0,0055 |
| Q2KJ25 | 26S proteasome non-ATPase regulatory subunit 12 | 0,745 | 0,0031 |
| P21809 | Biglycan | 0,744 | 0,0036 |
| Q3T0R1 | 40S ribosomal protein S18 | 0,743 | 0,0037 |
| Q5E995 | 40S ribosomal protein S6 | 0,742 | 0,0063 |
| F1N632 | Dolichyl-diphosphooligosaccharide--protein glycosyltransferase 48 kDa subunit | 0,742 | 0,0054 |
| F1MK70 | Uncharacterized protein | 0,740 | 0,0058 |
| Q3T171 | 60S ribosomal protein L36 | 0,738 | 0,0043 |
| A4FUY3 | Procollagen-proline 3-dioxygenase | 0,738 | 0,0027 |
| F1MYW7 | Histocompatibility minor 13 | 0,737 | 0,0049 |
| F1MN04 | AIR carboxylase | 0,737 | 0,0048 |
| E1BAJ4 | Starch binding domain 1 | 0,735 | 0,0029 |
| Q3SZF2 | ADP-ribosylation factor 4 | 0,732 | 0,0049 |
| F1MLE8 | SEC22 homolog B, vesicle trafficking protein | 0,732 | 0,0036 |
| Q24JZ4 | Metadherin | 0,731 | 0,0042 |
| E1BKW5 | SEC23 interacting protein | 0,731 | 0,0042 |
| Q08E58 | Tubulin tyrosine ligase like 12 | 0,729 | 0,0012 |
| Q3SZH7 | Leukotriene A-4 hydrolase | 0,724 | 0,0023 |
| A0A3Q1ME09 | Microtubule actin crosslinking factor 1 | 0,719 | 0,0029 |
| Q58DW0 | 60S ribosomal protein L4 | 0,718 | 0,0008 |
| Q08DQ2 | Glutamine--fructose-6-phosphate aminotransferase [isomerizing] 2 | 0,717 | 0,0025 |
| A0A3Q1LPB5 | 40S ribosomal protein S8 | 0,717 | 0,0014 |
| Q58DQ3 | 60S ribosomal protein L6 | 0,714 | 0,0012 |
| A0A3Q1LNH8 | Septin-8 | 0,714 | 0,0021 |
| Q2YDE4 | Proteasome subunit alpha type-6 | 0,714 | 0,0001 |
| P52193 | Calreticulin | 0,713 | 0,0011 |
| E1B970 | Golgin A3 | 0,713 | 0,0018 |
| Q3T0Z7 | Dihydropteridine reductase | 0,712 | 0,0017 |
| A1L504 | Lipase maturation factor 2 | 0,711 | 0,0006 |
| A0A3Q1M9B3 | 60S ribosomal protein L18 | 0,709 | 0,0016 |
| Q56JV9 | 40S ribosomal protein S3a | 0,704 | 0,0014 |
| Q0IIF7 | Ubiquitin carboxyl-terminal hydrolase 14 | 0,703 | 0,0008 |
| A0A3Q1MCJ7 | Uncharacterized protein | 0,701 | 0,0012 |
| Q5E9F1 | B-cell receptor-associated protein | 0,700 | 0,0006 |
| Q2KIL5 | Protein disulfide-isomerase A5 | 0,699 | 0,0012 |
| Q5E988 | 40S ribosomal protein S5 | 0,698 | 0,0009 |
| Q3SWX1 | Protein canopy homolog 4 | 0,696 | 0,0002 |
| F1MJQ4 | Procollagen-proline 4-dioxygenase | 0,695 | 0,0011 |
| A0A3Q1LTA5 | Ena/VASP-like protein | 0,694 | 0,0010 |
| A0A3Q1LUT2 | Eukaryotic translation initiation factor 4 gamma 1 | 0,694 | 0,0010 |
| P00570 | Adenylate kinase isoenzyme 1 | 0,693 | 0,0009 |
| P20000 | Aldehyde dehydrogenase, mitochondrial | 0,689 | 0,0008 |
| Q2TBR3 | SET nuclear oncogene | 0,688 | 0,0001 |
| Q5E9X4 | Leucine-rich repeat-containing protein 59 | 0,687 | 0,0006 |
| A0A3Q1LUG9 | Uncharacterized protein | 0,685 | 0,0001 |
| Q08E11 | Peptidyl-prolyl cis-trans isomerase C | 0,684 | 0,0001 |
| Q2KJH7 | Delta-1-pyrroline-5-carboxylate synthase | 0,683 | 0,0002 |
| A5D989 | Elongation factor 1-delta | 0,678 | 0,0005 |
| Q58D08 | Pyrroline-5-carboxylate reductase 3 | 0,678 | 0,0001 |
| Q2TBQ5 | 60S ribosomal protein L7a | 0,675 | 0,0004 |
| A0A3Q1M445 | Transmembrane 9 superfamily member | 0,672 | 0,0004 |
| Q5E946 | Parkinson disease protein 7 homolog | 0,672 | 0,0002 |
| Q58DT4 | Pyrroline-5-carboxylate reductase 1, mitochondrial | 0,670 | 0,0002 |
| P26452 | 40S ribosomal protein SA | 0,668 | 0,0002 |
| A0A3Q1MMW5 | 60S ribosomal protein L5 | 0,668 | 0,0003 |
| F1MRE5 | Acyl-CoA binding domain containing 3 | 0,668 | 0,0002 |
| A0A3Q1MKJ7 | Glucosidase 2 subunit beta | 0,667 | 0,0002 |
| A0A3Q1NC75 | Calumenin | 0,667 | 0,0001 |
| Q3SYV9 | ADP-ribose glycohydrolase ARH3 | 0,667 | 0,0001 |

**Table S3. Table of proteins without statistically significant changes between CD44+ and CD44- cells.**

| **Uniprot ID** | **Name** |
| --- | --- |
| A0A140T832 | 40S ribosomal protein S17 |
| A0A140T856 | Coatomer subunit alpha |
| A0A140T861 | RNA-binding protein FUS |
| A0A140T886 | Coatomer subunit gamma |
| A0A140T894 | 14-3-3 protein beta/alpha |
| A0A140T8A5 | Isocitrate dehydrogenase [NADP] |
| A0A3Q1LK06 | LRR binding FLII interacting protein 1 |
| A0A3Q1LKR8 | Ubiquitin-activating enzyme E1 |
| A0A3Q1LM31 | Far upstream element binding protein 1 |
| A0A3Q1LPX4 | 3-hydroxy-3-methylglutaryl coenzyme A synthase |
| A0A3Q1LQI5 | Eukaryotic translation initiation factor 2 subunit 2 |
| A0A3Q1LR67 | Uncharacterized protein |
| A0A3Q1LRD1 | Phosphoglucomutase-1 |
| A0A3Q1LSB6 | ATP-citrate synthase |
| A0A3Q1LUJ8 | Sodium/potassium-transporting ATPase subunit alpha |
| A0A3Q1LVC8 | Xaa-Pro aminopeptidase 1 |
| A0A3Q1LVS2 | Kinectin 1 |
| A0A3Q1LW82 | N-myc downstream-regulated gene 1 protein |
| A0A3Q1LXR2 | Ras-related C3 botulinum toxin substrate 1 |
| A0A3Q1M0D3 | Hepatoma-derived growth factor |
| A0A3Q1M3N4 | Unconventional myosin-Ic |
| A0A3Q1M4M4 | ATP synthase F(0) complex subunit B1, mitochondrial |
| A0A3Q1M722 | Aldehyde dehydrogenase |
| A0A3Q1M8R8 | Transmembrane protein 263 |
| A0A3Q1ME53 | 60S ribosomal protein L9 |
| A0A3Q1MFL7 | Actin-depolymerizing factor |
| A0A3Q1MH46 | Trifunctional enzyme subunit beta, mitochondrial |
| A0A3Q1ML66 | ATP synthase membrane subunit f |
| A0A3Q1MLQ7 | Talin 1 |
| A0A3Q1MME4 | Heterogeneous nuclear ribonucleoproteins A2/B1 |
| A0A3Q1MMQ9 | Activated RNA polymerase II transcriptional coactivator p15 |
| A0A3Q1MPG8 | 1,4-alpha-glucan branching enzyme |
| A0A3Q1MQ24 | RAN binding protein 3 |
| A0A3Q1MTI8 | 40S ribosomal protein S25 |
| A0A3Q1MVM8 | ATP synthase peripheral stalk subunit OSCP |
| A0A3Q1MVT2 | Serine/arginine-rich splicing factor 1 |
| A0A3Q1MVZ1 | Poly [ADP-ribose] polymerase |
| A0A3Q1MXU7 | Spectrin beta chain |
| A0A3Q1N0N9 | SEC31 homolog A, COPII coat complex component |
| A0A3Q1N348 | Heterogeneous nuclear ribonucleoprotein H1 |
| A0A3Q1N6T1 | NADH-cytochrome b5 reductase |
| A0A3Q1N8I6 | Phosphatidylinositol binding clathrin assembly protein |
| A0A3Q1NE82 | AHNAK nucleoprotein |
| A0A3Q1NKD1 | ELKS/RAB6-interacting/CAST family member 1 |
| A0A3S5ZPF7 | Phosphoserine aminotransferase |
| A0A3S5ZPM3 | 6-phosphogluconate dehydrogenase, decarboxylating |
| A0A452DI03 | Pre-B-cell leukemia transcription factor-interacting protein 1 |
| A0A452DI87 | Succinate dehydrogenase [ubiquinone] iron-sulfur subunit, mitochondrial |
| A1L5B7 | SERPINE1 mRNA binding protein 1 |
| A3KN04 | Dolichyl-diphosphooligosaccharide--protein glycosyltransferase subunit 1 |
| A4FV37 | Caveolae-associated protein 3 |
| A4IFT6 | TMED7 protein |
| A5D7A2 | Diadenosine tetraphosphate synthetase |
| A5D7E8 | Protein disulfide-isomerase |
| A5D984 | Pyruvate kinase |
| A5D9H5 | Heterogeneous nuclear ribonucleoprotein D |
| A5PJZ1 | Calcium-binding mitochondrial carrier protein SCaMC-1 |
| A6QQR5 | TMEM43 protein |
| A7E3Q2 | Heat shock 70kDa protein 1A |
| A7E3W7 | ATP-dependent RNA helicase DDX1 |
| A7MB21 | FERMT2 protein |
| A7MBA2 | 26S proteasome non-ATPase regulatory subunit 1 |
| A7MBJ5 | Cullin-associated NEDD8-dissociated protein 1 |
| E1B8K6 | Nucleolin |
| E1BB38 | Signal recognition particle subunit SRP72 |
| E1BG25 | Melanotransferrin |
| E1BG76 | Sec1 family domain containing 1 |
| E1BGB0 | Kinesin family member 13B |
| E1BGJ0 | LDL receptor related protein 1 |
| E1BKM4 | Programmed cell death 6 interacting protein |
| E1BMW9 | Purine rich element binding protein A |
| E1BN47 | Cytoplasmic FMR1-interacting protein |
| E1BNE7 | Caveolae associated protein 1 |
| E1BP91 | Aminopeptidase |
| F1MG05 | Elongation factor 1-gamma |
| F1MIU2 | BAG cochaperone 3 |
| F1MJH1 | Actin-depolymerizing factor |
| F1MMA0 | Interleukin enhancer binding factor 3 |
| F1MR96 | Calpain inhibitor |
| F1MTY3 | Helix-destabilizing protein |
| F1MUN7 | Annexin |
| F1MUP9 | Vesicle amine transport 1 |
| F1MYG5 | Lamin A/C |
| F1N0J2 | Microtubule-associated protein |
| F1N3V0 | Malic enzyme |
| F1N647 | 3-hydroxyacyl-[acyl-carrier-protein] dehydratase |
| F1N6Y7 | Eukaryotic translation initiation factor 5B |
| F6PS38 | Y-box binding protein 3 |
| F6PWM7 | Aminoacyl tRNA synthetase complex interacting multifunctional protein 1 |
| F6QJJ8 | Progesterone receptor membrane component 2 |
| F6RFP6 | Basigin |
| G3MXC8 | Actin-related protein 2/3 complex subunit 5 |
| G3MYE7 | Methyl-CpG-binding protein 2 |
| G3X6L9 | Glutamyl-tRNA synthetase |
| G5E531 | CCT-alpha |
| G5E589 | Proteasome subunit beta |
| G8JKX8 | Coatomer subunit beta' |
| O02675 | Dihydropyrimidinase-related protein 2 |
| O62768 | Thioredoxin reductase 1, cytoplasmic |
| O77834 | Peroxiredoxin-6 |
| P00727 | Cytosol aminopeptidase |
| P00829 | ATP synthase subunit beta, mitochondrial |
| P02584 | Profilin-1 |
| P04896 | Guanine nucleotide-binding protein G(s) subunit alpha isoforms short |
| P10096 | Glyceraldehyde-3-phosphate dehydrogenase |
| P10103 | High mobility group protein B1 |
| P10881 | Lupus La protein homolog |
| P12344 | Aspartate aminotransferase, mitochondrial |
| P12378 | UDP-glucose 6-dehydrogenase |
| P13214 | Annexin A4 |
| P19120 | Heat shock cognate 71 kDa protein |
| P19803 | Rho GDP-dissociation inhibitor 1 |
| P21856 | Rab GDP dissociation inhibitor alpha |
| P28801 | Glutathione S-transferase P |
| P30932 | CD9 antigen |
| P33097 | Aspartate aminotransferase, cytoplasmic |
| P35466 | Protein S100-A4 |
| P50397 | Rab GDP dissociation inhibitor beta |
| P53619 | Coatomer subunit delta |
| P55859 | Purine nucleoside phosphorylase |
| P62248 | Myeloid-derived growth factor |
| P62935 | Peptidyl-prolyl cis-trans isomerase A |
| P62992 | Ubiquitin-40S ribosomal protein S27a |
| P63103 | 14-3-3 protein zeta/delta |
| P63243 | Receptor of activated protein C kinase 1 |
| P68002 | Voltage-dependent anion-selective channel protein 2 |
| P68103 | Elongation factor 1-alpha 1 |
| P68252 | 14-3-3 protein gamma |
| P68509 | 14-3-3 protein eta |
| P80311 | Peptidyl-prolyl cis-trans isomerase B |
| P80513 | Mesencephalic astrocyte-derived neurotrophic factor |
| Q05588 | Urokinase plasminogen activator surface receptor |
| Q08D83 | Reticulon-3 |
| Q0IIF7 | Ubiquitin carboxyl-terminal hydrolase 14 |
| Q0VCK0 | Bifunctional purine biosynthesis protein ATIC |
| Q0VCM4 | Glycogen phosphorylase, liver form |
| Q17QE2 | LIM and cysteine-rich domains protein 1 |
| Q17R07 | ADP-ribosylation factor GTPase-activating protein 3 |
| Q1RMM9 | Alpha-galactosidase |
| Q1RMR3 | Cortactin |
| Q1RMX7 | N-acetylneuraminate synthase |
| Q27991 | Myosin-10 |
| Q28056 | Aspartyl/asparaginyl beta-hydroxylase |
| Q28141 | ATP-dependent RNA helicase A |
| Q29RV1 | Protein disulfide-isomerase A4 |
| Q2HJ33 | Obg-like ATPase 1 |
| Q2HJ49 | Moesin |
| Q2HJ81 | Tubulin beta-6 chain |
| Q2HJ89 | Peptidyl-prolyl cis-trans isomerase FKBP10 |
| Q2KI42 | 26S proteasome non-ATPase regulatory subunit 11 |
| Q2KJ47 | EH-domain containing 2 |
| Q2KJ93 | Cell division control protein 42 homolog |
| Q2KJC8 | Peptidyl-prolyl cis-trans isomerase FKBP9 |
| Q2KJH9 | 4-trimethylaminobutyraldehyde dehydrogenase |
| Q2NKY7 | Septin-2 |
| Q2NL26 | Transketolase-like protein 1 |
| Q2TBX1 | Cytokine induced protein 29 kDa |
| Q32LG3 | Malate dehydrogenase, mitochondrial |
| Q32PB9 | 60S ribosomal protein L38 |
| Q3MHH4 | Glutamine--tRNA ligase |
| Q3MI00 | DnaJ homolog subfamily B member 1 |
| Q3SX33 | Thy-1 cell surface antigen |
| Q3SYU2 | Elongation factor 2 |
| Q3SZ62 | Phosphoglycerate mutase 1 |
| Q3SZ65 | Eukaryotic initiation factor 4A-II |
| Q3SZC4 | NSFL1 cofactor p47 |
| Q3SZI0 | Mannose-6-phosphate isomerase |
| Q3T054 | GTP-binding nuclear protein Ran |
| Q3T0D5 | 60S ribosomal protein L30 |
| Q3T0K2 | T-complex protein 1 subunit gamma |
| Q3T0L2 | Endoplasmic reticulum resident protein 44 |
| Q3T0Q4 | Nucleoside diphosphate kinase B |
| Q3T0U1 | LRP chaperone MESD |
| Q3T0X5 | Proteasome subunit alpha type-1 |
| Q3T0Y5 | Proteasome subunit alpha type-2 |
| Q3T108 | Proteasome subunit beta type-4 |
| Q3T145 | Malate dehydrogenase, cytoplasmic |
| Q3T147 | Spliceosome RNA helicase DDX39B |
| Q3T149 | Heat shock protein beta-1 |
| Q3T160 | Nucleophosmin |
| Q3ZBD7 | Glucose-6-phosphate isomerase |
| Q3ZBG0 | Proteasome subunit alpha type-7 |
| Q3ZBH0 | T-complex protein 1 subunit beta |
| Q3ZBL4 | Leucine zipper transcription factor-like protein 1 |
| Q3ZBM5 | Sorting nexin-5 |
| Q3ZBT5 | Syntaxin-7 |
| Q3ZBV8 | Threonine--tRNA ligase 1, cytoplasmic |
| Q3ZCD0 | CD81 antigen |
| Q3ZCF5 | Ornithine aminotransferase, mitochondrial |
| Q3ZCH0 | Stress-70 protein, mitochondrial |
| Q3ZCI9 | T-complex protein 1 subunit theta |
| Q3ZCJ2 | Aldo-keto reductase family 1 member A1 |
| Q3ZCK9 | Proteasome subunit alpha type-4 |
| Q56JX3 | 60S ribosomal protein L31 |
| Q5E947 | Peroxiredoxin-1 |
| Q5E956 | Triosephosphate isomerase |
| Q5E971 | Transmembrane emp24 domain-containing protein 10 |
| Q5E9A1 | Nascent polypeptide-associated complex subunit alpha |
| Q5E9B7 | Chloride intracellular channel protein 1 |
| Q5E9E6 | 60S ribosomal protein L10a |
| Q5E9F2 | Heme oxygenase 1 |
| Q5E9F5 | Transgelin-2 |
| Q5E9F7 | Cofilin-1 |
| Q5E9F9 | 26S proteasome regulatory subunit 7 |
| Q5E9R3 | EH domain-containing protein 1 |
| Q5E9T9 | RNA-splicing ligase RtcB homolog |
| Q76LV2 | Heat shock protein HSP 90-alpha |
| Q863B3 | Staphylococcal nuclease domain-containing protein 1 |
| Q8MJG1 | PC4 and SFRS1-interacting protein |
| Q8WN55 | Polypyrimidine tract-binding protein 1 |
| Q95L54 | Annexin A8 |
| Q95M18 | Endoplasmin |
| Q9GMB8 | Serine--tRNA ligase, cytoplasmic |
| Q9XSA7 | Chloride intracellular channel protein 4 |
| Q9XSI3 | 60S ribosomal protein L10 |
